# A Novel Flexure-Based Hybrid Mechanism for Micropositioning: Design, Analysis and Experimental Verification

**DOI:** 10.3390/s26216854

**Published:** 2026-10-29

**Authors:** Kim Sang Tran, Bijan Shirinzadeh, Zexuan Hu, Yanding Qin, Yongmin Zhong, Julian Smith, Dhanya Menoth Mohan, Wessam Salem

**Affiliations:** 1 Robotics and Mechatronics Research Laboratory, Department of Mechanical and Aerospace Engineering, Monash University, Melbourne, VIC 3800, Australia; kim.tran4@monash.edu (K.S.T.); hu.hu@monash.edu (Z.H.); dhanya.menothmohan1@monash.edu (D.M.M.); wessam.salem1@monash.edu (W.S.); 2 College of Computer and Control Engineering, Nankai University, Tianjin 300350, China; qinyd@nankai.edu.cn; 3 School of Engineering, RMIT University, Bundoora, VIC 3083, Australia; yongmin.zhong@rmit.edu.au; 4 Department of Surgery, Monash University, Melbourne, VIC 3168, Australia; julian.smith@monash.edu

**Keywords:** hybrid mechanism, dual-stage actuation, laser-based sensing and measurement, long-range micropositioning, flexure mechanism

## Abstract

Flexure mechanisms, precision actuation, and micro/nanoscale sensing and control are the fundamental building blocks for precision engineering, micro/nanopositioning systems, and instrumentation for micro/nanoscale manufacturing. Furthermore, a growing number of applications require both millimeter-scale workspace range and nanometer-scale resolution from a single positioning stage. However, achieving simultaneous long-range displacement and nanometer-scale precision positioning remains a significant challenge in micropositioning systems. This paper presents a novel hybrid flexure-based mechanism that integrates a voice coil motor (VCM) for a millimeter-scale positioning of a long-range motion stage, with a piezoelectric actuator (PEA) for the high-resolution positioning stage. The two stages are not connected directly: the piezoelectric actuator drives a bridge-type displacement amplifier whose output reaches the platform through decoupling parallelogram flexures, mechanically isolating the precision motion stage from the long-range motion input. Under closed-loop control, the coupled displacement of the hybrid motion is approximately 0.15%, compared with about 0.3% when the long-range stage is driven alone by the VCM, improving positional accuracy and stability during combined motion. This architecture provides a continuous transition between coarse and fine motion positioning without exposing the piezoelectric stack to adverse loading effects. Analytical modeling, finite element analysis, and experimental validation show that the system delivers a travel range of 2 mm with a resolution of 10 nm in the long-range stage and 5 nm in the high-precision stage. Experimental results show that the hybrid mode improves sensing and control specification for the rise time from 32.40 ms to 18.90 ms, the settling time from 51.90 ms to 32.10 ms, and the overshoot from 24.65% to 5.70% relative to long-range motion actuation alone.

## 1. Introduction

Demand for flexure-based micropositioning mechanisms has grown rapidly for precision engineering applications such as microfabrication and biomedical instrumentation [1,2]. Such mechanisms are the key basis to achieve high-precision positioning and measurement over limited working space. Micropositioning mechanisms are typically built from flexure beams and flexure hinges, and are driven by piezoelectric actuators (PEAs), often with an amplification structure [3]. However, their output displacement remains limited. To address this drawback, long-range mechanisms driven by voice coil motors (VCMs) have been proposed to generate larger input displacements, achieving extended travel range, but typically with sacrifices in resolution [4].

Many applications require a single stage to deliver both long-range travel and high-precision motion. Applications in micro/nanotechnology [5,6], biomedical devices and instrumentation [7], micro/nanoscale imaging [8,9,10], and precision manufacturing [11,12] require systems that can perform complex tasks with high accuracy [13,14]. Flexure-based mechanisms have been used to transfer the input motion to the output displacement with high precision. However, despite their numerous advantages, flexure-based mechanisms alone may not be sufficient for applications requiring large motion ranges. This limitation has led to the exploration of hybrid mechanisms that combine flexure elements with other actuator types to attain high precision together with a long motion range. Previous studies have shown that linking a VCM and a PEA through a hybrid flexure-based bridge enables a system to deliver both long-range displacement and high positioning precision [15,16].

Voice coil motors share a well-established capability as actuators with long-range displacement. VCMs are electromagnetic actuators known for direct-drive linear motion, high precision, and rapid response. Their operation is based on the Lorentz force, in which a current-carrying conductor placed in a magnetic field experiences a force that produces linear motion [17]. VCMs are widely used in precision applications such as hard disk drives and optical devices due to their simple construction, high efficiency, and ability to deliver significant force over relatively long ranges [18,19]. The linear motion characteristics of VCMs make them suitable for long-stroke actuation [20], complementing the precision capabilities of flexure mechanisms and piezoelectric actuators.

Piezoelectric actuators, by contrast, offer high precision and fast response. They are well suited to applications requiring high-resolution motion control, such as micropositioning and vibration suppression [21]. Piezoelectric actuators operate through the piezoelectric effect, in which a piezoelectric material experiences deformation in response to an applied electric field, resulting in precise motion. Although piezoelectric actuators can achieve sub-nanometer resolution, their motion range is typically limited [22,23,24,25,26]. The above characteristics of VCM and PEA provide the motivation for the development of hybrid mechanisms that combine such actuator types to exploit their respective strengths in motion range and resolution.

Several dual-stage flexure mechanisms have been reported. Research efforts have sought to couple a VCM and a PEA directly using a frictional constraint between the two stages [27]. Researchers integrated a VCM coarse stage and a PEA fine stage in parallel through an A-shaped compliant mechanism, reaching a travel range of about 1.5 mm, although interference between the long-range and precision motions remained [28]. Other research efforts led to the development of a hybrid nanopositioner for atomic force microscopy in which normal-stressed electromagnetic actuators drive the xy stage and a PEA drives the z axis; the travel displacement was limited to 103 μm [29]. Other researchers presented a linear-motion dual-stage actuation system using a VCM and a PEA mounted inside a housing, achieving a range of 30 μm [30]. In each of the above designs the piezoelectric-based motion stage is either loaded directly by the coarse motion stage or constrained to a fixed base, which limits the long-range motion input that can be applied. This paper presents a hybrid mechanism in which the VCM and PEA are linked through a bridge-type amplifier and decoupling flexure beams. It is proposed that a millimeter-scale motion range can be combined with nanometer-scale motion resolution.

The development of hybrid flexure-based mechanisms involves several key processes, including the mechanical design of the flexure-based structure, optimization and analysis of actuator integration, and a sensing and control strategy. The flexure structure must be carefully designed to ensure that it can handle both long-range coarse motion and fine motion without introducing significant coupled motions or nonlinearities [31,32]. The hybrid mechanisms described above remain constrained by the lack of their long-range travel, and by the direct connection between the VCM and the PEA. Such a connection transmits long-range motion loading into the piezoelectric stack, and thus can fracture its layers, while a fixed long-range motion stage restricts the input motion available to the first stage.

This paper addresses these important aspects with a flexure-based hybrid micropositioning mechanism. The entire frame of long-range motion stage is carried on bearing slider blocks, which allows a millimeter-scale input displacement to be applied by the VCM. The piezoelectric actuator is not connected directly to the VCM; instead, it drives a bridge-type flexure amplifier whose output is transmitted to the compliant motion stage through decoupling parallelogram beams. This separation protects the piezoelectric stack from the potential excessive loading of the long-range motion platform, while the decoupling beams minimize coupled motion of the platform. The main contributions of this work are as follows: First, a two-stage flexure architecture is proposed in which the coarse and fine motion stages are mechanically decoupled by a bridge-type displacement amplifier and parallelogram flexures. The above removes the risks of piezoelectric layer fracture under long-range input and allows the long-range frame to travel on bearing slider blocks. Second, closed-form analytical models for the displacement amplification ratio and the input and output stiffnesses are derived using Castigliano’s second theorem and beam constraint theory. A lumped-parameter dynamic model of the long-range motion stage is also developed. The analytical models are verified with the computational finite element analysis (FEA) results, and errors are identified. Third, sensing and actuation techniques, together with an experimental validation of a 2 mm travel range with resolutions of 10 nm in the long-range motion stage and 5 nm in the precision stage, are established. The experiments reveal a reduction of the coupled displacement from 0.3% under long-range motion actuation alone to 0.15% when both stages operate together under PID control. This work is significant because it shows that millimeter-scale travel and nanometer-scale resolution can be obtained from a hybrid mechanism while keeping the coarse and fine stages from interfering with each other, an interaction that limits the performance of existing dual-stage positioning systems.

The remainder of this paper is organized as follows. Section 2 presents the design and analytical modeling of the hybrid mechanism. Section 3 describes the computational analysis. Section 4 presents the experimental system identification and results. Section 5 concludes the paper.

## 2. Design of Hybrid Flexure-Based Mechanism

### 2.1. Coupling Motion Analysis

Flexure-based beams were designed to connect the long-range motion stage and the precision motion stage. For the first stage, a VCM was chosen to generate the long-range input displacement via the coil of the VCM, which is connected to the frame positioned on bearing sliders so that the long-range displacement can be transferred directly from the VCM to the entire mechanism. For the second stage, a piezoelectric actuator was selected to drive the precision input displacement, and it was designed to be mounted inside an amplification bridge-type mechanism. This amplification mechanism was then connected to a platform via the decoupling parallelogram beams that were aimed to minimize the coupled motion for this hybrid mechanism. An end-effector was positioned on the platform to reflect the motions of the proposed hybrid mechanism.

**Figure 1:**
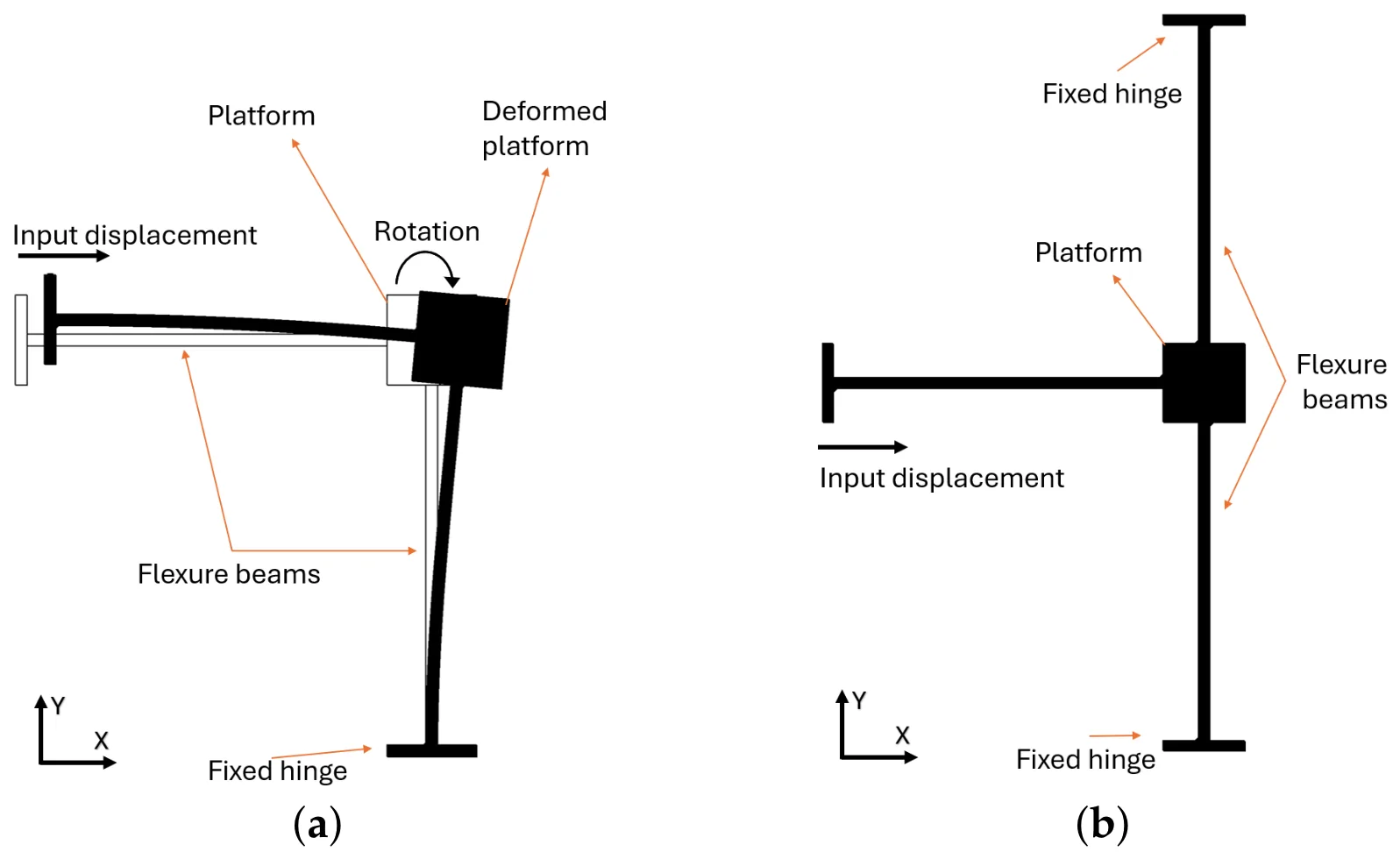
Working principle of (**a**) the two-flexure-beam mechanism; (**b**) the three-flexure-beam mechanism.

A platform is normally attached to two flexure beams, as shown in Figure 1a. When an input displacement is applied along the X-direction, in addition to linear displacement, rotary motion will also occur, which is considered as a coupling motion, while the desired output motion is only linear displacement. Therefore, another flexure beam was presented to support the coupling analysis, as shown in Figure 1b.

**Figure 2:**
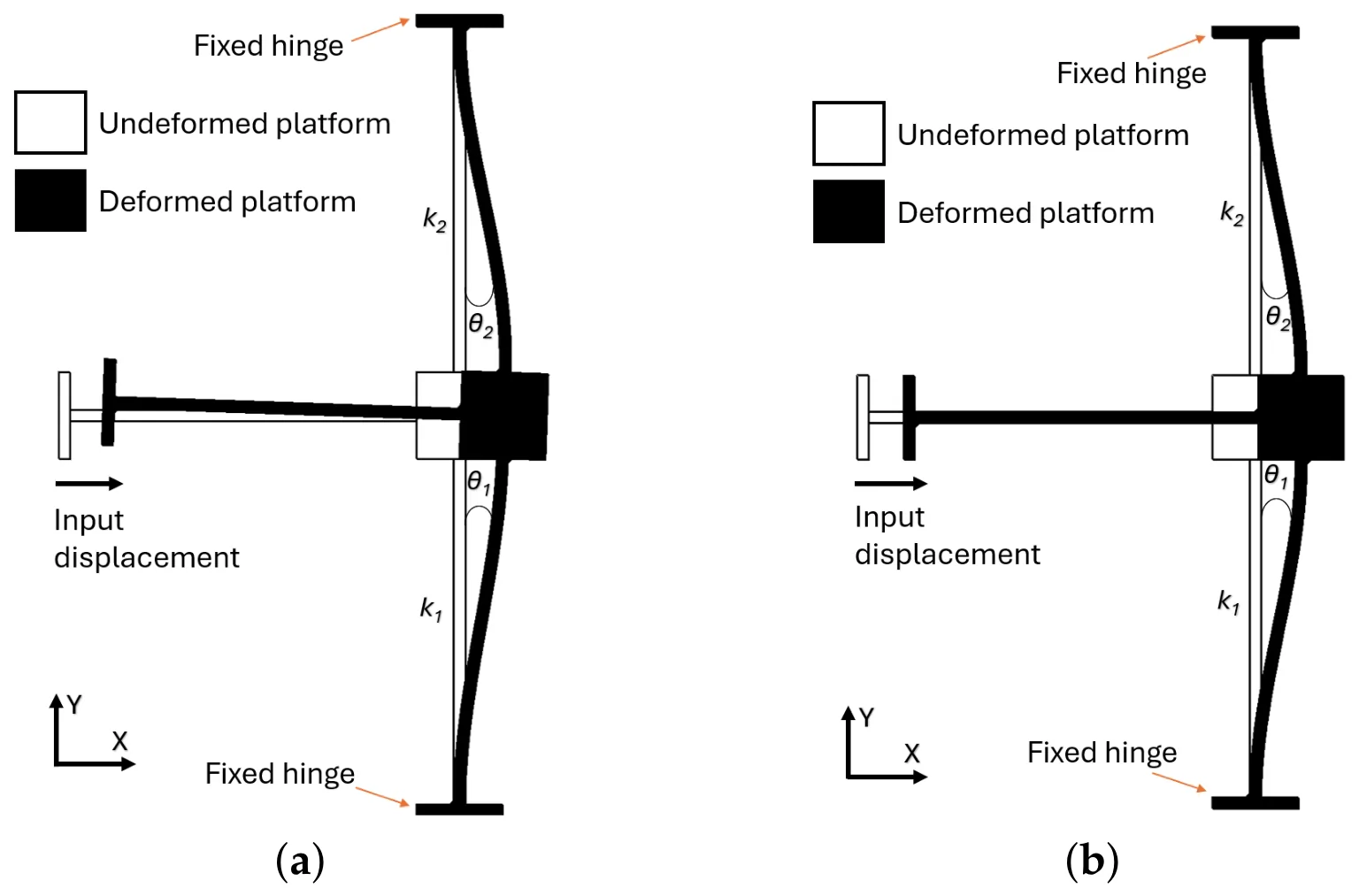
Two-flexure-beam mechanism with (**a**) different stiffnesses; (**b**) equal stiffnesses in the two flexure beams.

Figure 2 presents a platform with two flexure beams in parallel with the Y-direction, while the input displacement was designed in the X-direction. $k_{1}$ and $k_{2}$ are the stiffnesses of the two flexure beams, and $k_{in}$ is the input stiffness. $\theta_{1}$ and $\theta_{2}$ are the rotational angles caused by the input displacement. When an input displacement is applied to the platform, if $k_{1}<k_{2}$ then $\theta_{1}>\theta_{2}$, creating a coupled rotary motion, and vice versa, as shown in Figure 2a. If $k_{1}=k_{2}$, then $\theta_{1}=\theta_{2}$. As a result, only linear displacement is obtained, and the coupled rotary motion is eliminated. Therefore, the decoupling flexure structure was designed with $k_{1}=k_{2}$, as shown in Figure 2b.

**Figure 3:**
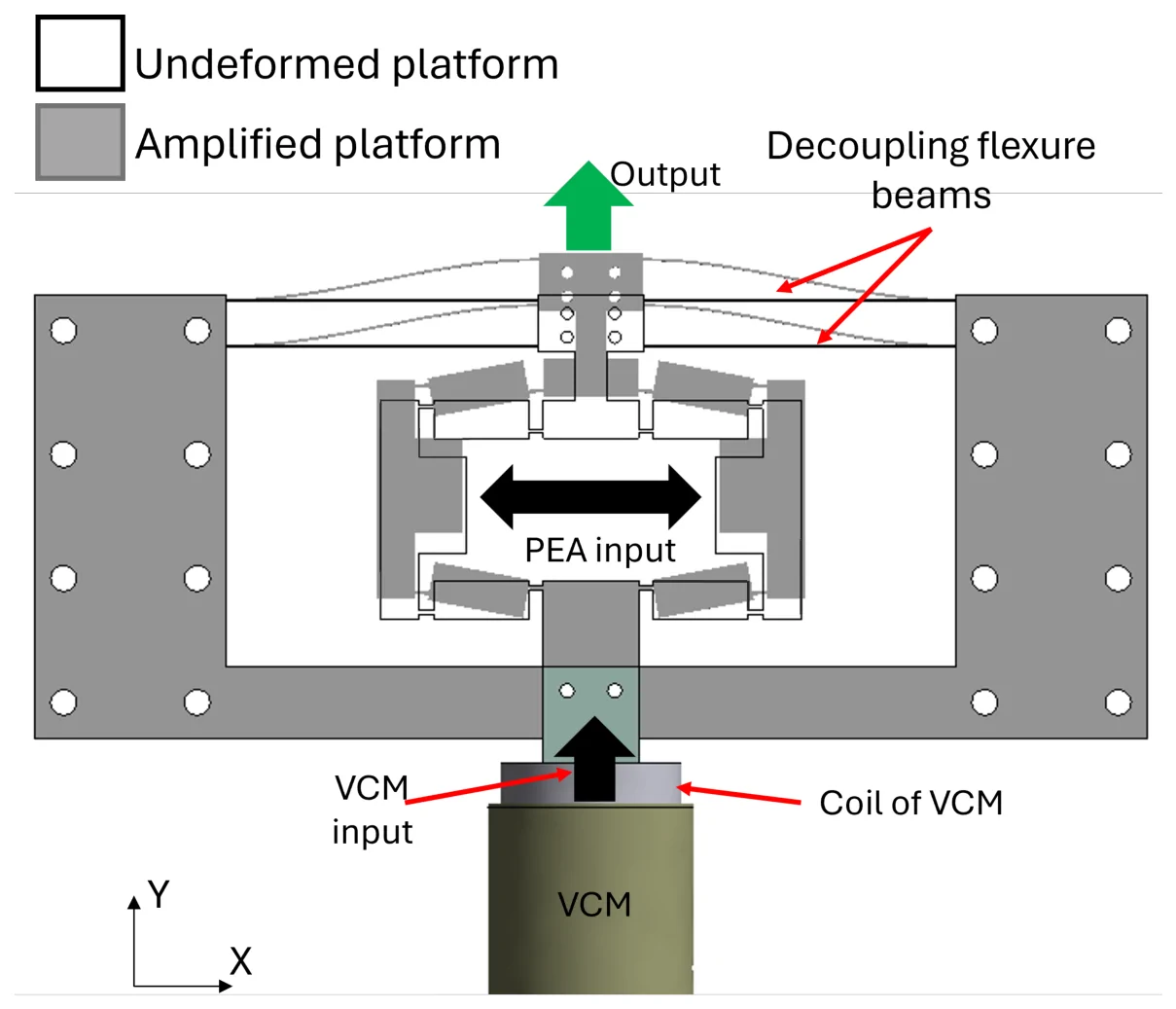
Amplification structure and decoupling parallelogram beams.

The proposed hybrid mechanism was designed with two stages, including the long-range and precision stages. The driving actuators were selected to generate the motions for this hybrid mechanism, and they were linked to each other by a flexure-based amplification structure and decoupling parallelogram beams. The right-angle notches were used to build the amplification structure, and then to provide for the high amplification ratio [33,34]. A voice coil motor was utilized to generate the long-range movement. The long-range stage was designed to reach the millimeter-scale motion, and a piezoelectric actuator was attached to the amplification mechanism, as shown in Figure 3.

**Figure 4:**
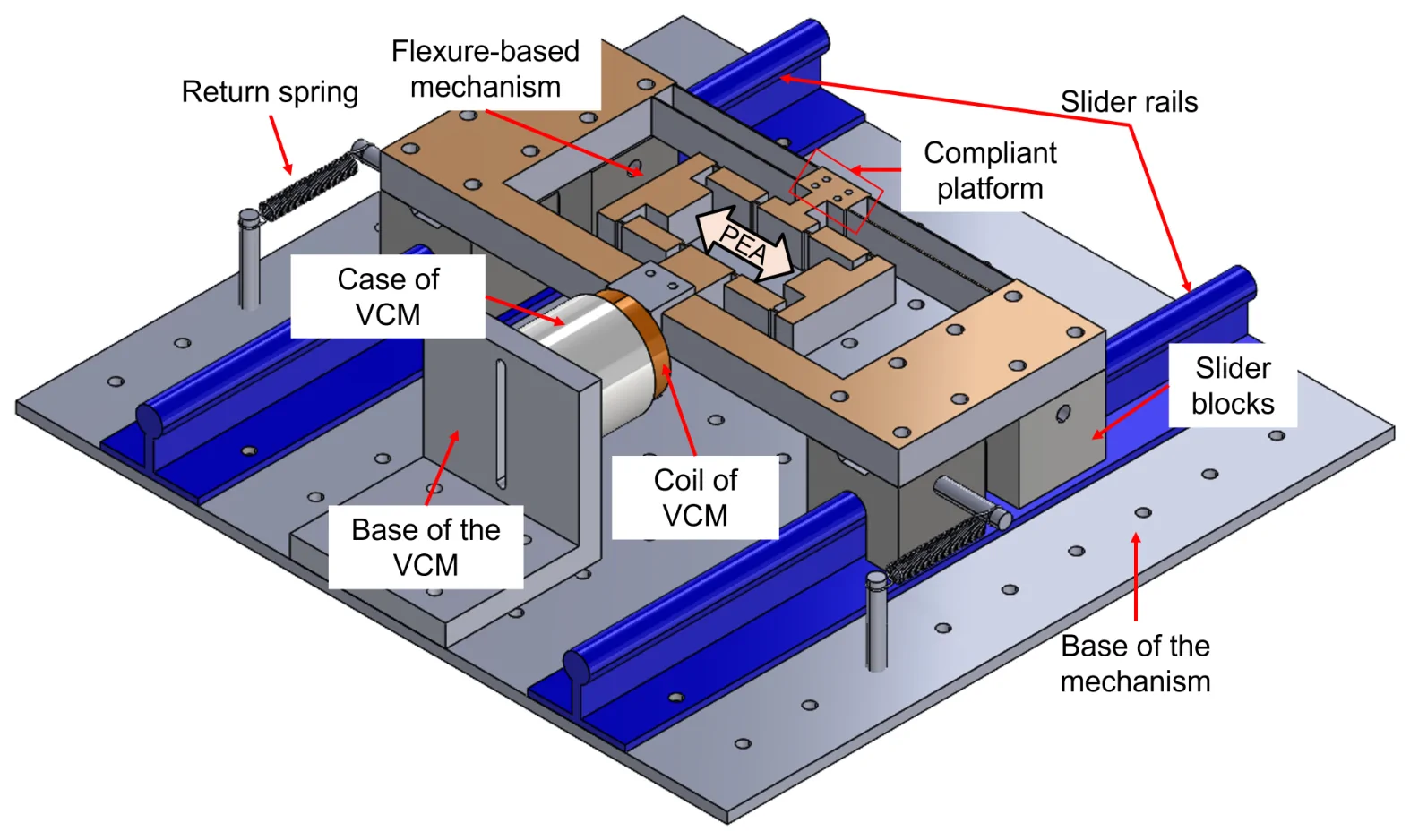
The proposed flexure-based hybrid mechanism.

One side of the amplification structure was connected to the large frame sitting on the slider blocks, where a coil of the VCM was attached. The other side of the amplification structure was linked to the stage with compliant mechanism and the flexure-based parallelogram structure, which offers linear deformation due to the amplification flexure principle, as indicated in Figure 4. In the proposed hybrid mechanism, the entire mechanism was placed on the slider blocks, and it was attached to the return springs. When the VCM is activated, the entire mechanism moves forward and backward, providing the long-range stage. The piezoelectric stack actuator is then energized to deliver precision displacement and high-resolution motion.

**Figure 5:**
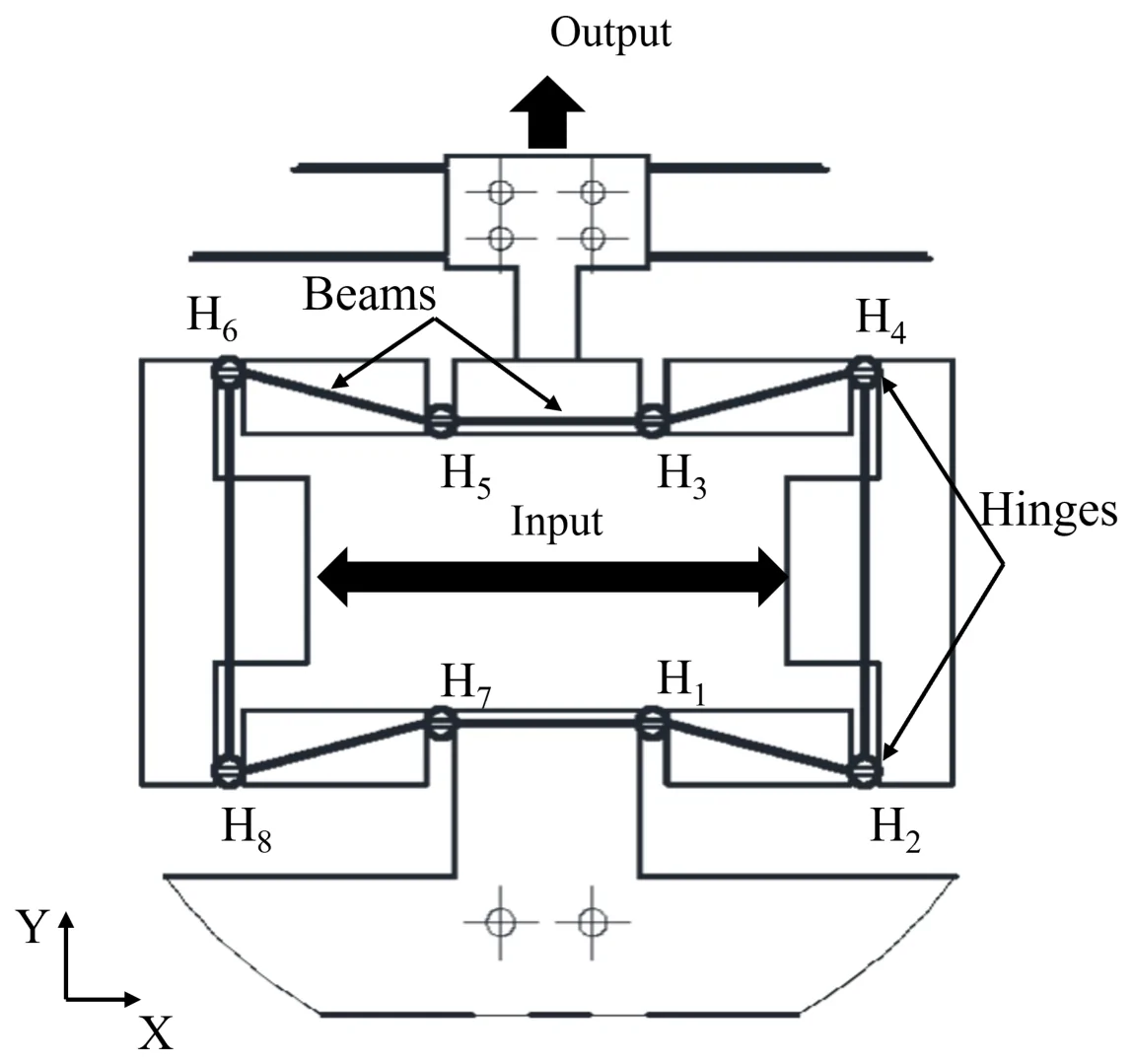
Working principle of flexure-based amplification mechanism.

In this proposed mechanism, only the Y-direction performance is described owing to the symmetry of this flexure structure. Therefore, only one branch is selected to show the working principle of this amplification mechanism. The overall strategy for the amplification principle is presented in the schematic diagram, as shown in Figure 5. When the piezoelectric actuator provides an input displacement $x_{input}$ along the X-axis (±ve), the corresponding output will be $2y_{output}$ along the Y-axis (±vc). In other words, when $H_{2}$ hinge moves $x_{input}$ in the positive X-direction, the $H_{1}$ hinge will move $y_{output}$ along the negative Y-direction. However, $H_{1}$ is fixed so that $H_{2}$ hinge will move $y_{output}$ along the positive Y-direction whilst moving $x_{input}$ along the X-axis. When hinge $H_{4}$ moves $x_{input}$ in the positive X-direction, hinge $H_{3}$ travels $y_{output}$ along the Y direction. In the meantime, $H_{2}$ also moves a distance of $y_{output}$ in the same direction as hinge $H_{3}$; therefore, the total output at hinge $H_{3}$ is $2y_{output}$.

Since this flexure mechanism is symmetrical, a similar principle applies to hinges $H_{5}$, $H_{6}$, $H_{7}$, and $H_{8}$. It is necessary to note that hinge $H_{3}$ and $H_{5}$ generate the same output, so the total output of this mechanism is $2y_{output}$ in the Y-direction. Furthermore, the beam $H_{3}H_{5}$ is connected to the decoupling parallelogram flexure beams, which have prismatic deformation along the Y-axis due to low transverse stiffness. Hence, the total output displacement can be monitored, as described in Figure 5.

### 2.2. Flexure-Based Amplification Mechanism

**Figure 6:**
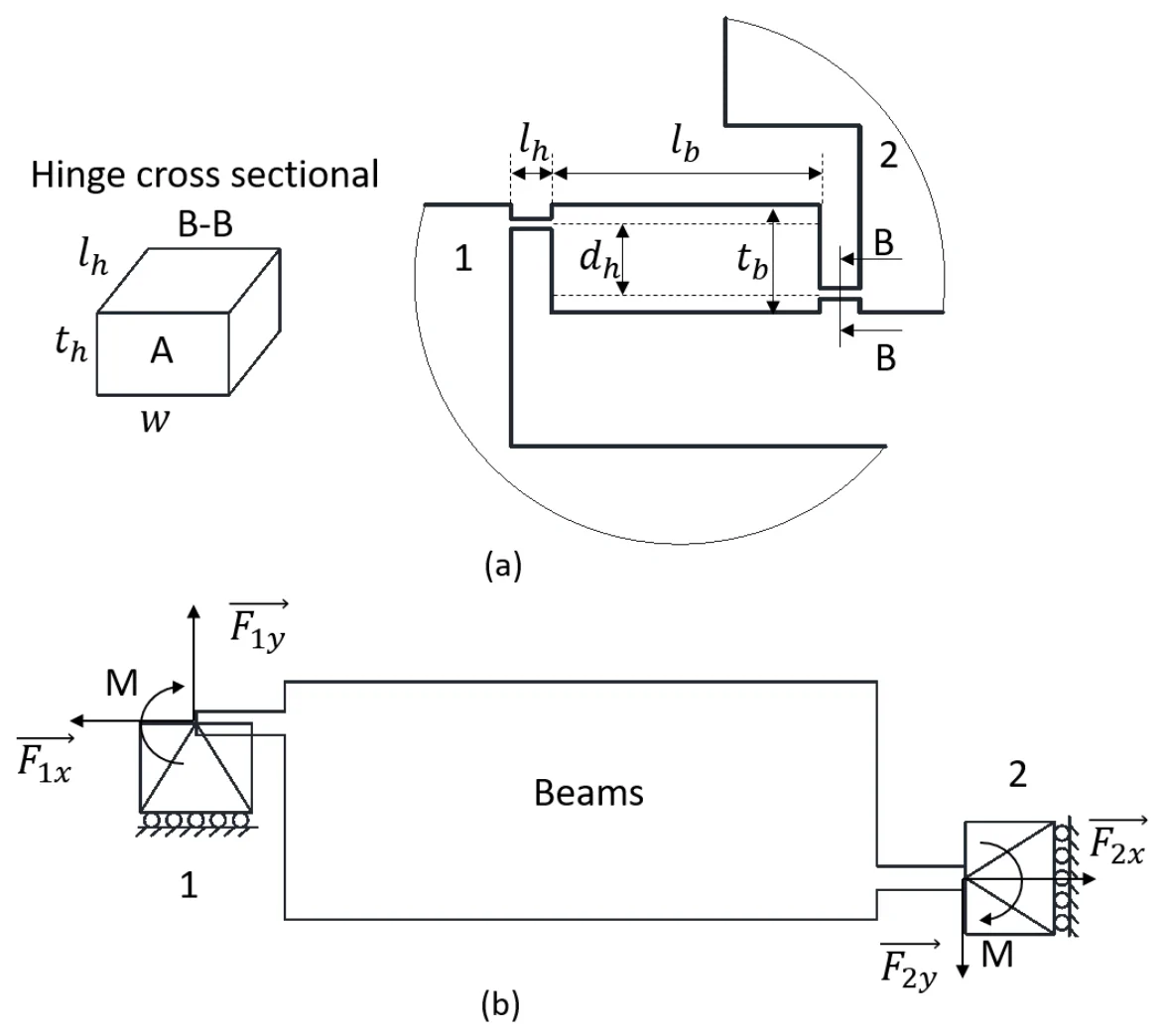
Analysis of amplification mechanism: (**a**) flexure hinge, (**b**) Flexure beam. (Labels 1 and 2 denote the two hinges of one branch of the flexure beam, *A* is the cross-sectional area of the flexure beam, B–B is the cross-sectional view of the hinge, and *M* is the moment at each hinge.)

Response surface analysis was performed with a safety factor of 2, and the optimized parameters for the proposed hybrid mechanism were obtained, as shown in Table 1. The amplification mechanism consists of flexure beams that are symmetrically arranged. Therefore, calculations are required for only one branch. Figure 6 indicates the equivalent model of a flexure beam connected to two flexible hinges at both ends. Since both hinges undergo the same deformation, it is assumed that the moments on both sides of the beam are equal. Based on the equations of moment and force, the following relationships can be derived:

(1) $$F_{1x}=F_{2x}=\frac{1}{2}F_{Piezo}=F_{x}$$

(2) $$F_{1y}=F_{2y}=\frac{1}{2}F_{Out}=F_{y}$$

(3) $$M_{1}+M_{2}=2M=F_{x}d_{h}-F_{y}\left(l_{h}+l_{b}\right)$$

When the piezoelectric actuator is activated to provide the input displacement, a corresponding relative output displacement is also observed through the amplification mechanism. According to Castigliano’s second theorem, the input $x_{input}$ and $y_{output}$ are identified by the following relationships:

(4) $$\begin{array}{cc} x_{input}= & 2\int_{0}^{l_{h}}\frac{F_{l}\left(x\right)}{EA_{h}}\;A\cdot d\left(x\right)+2\int_{0}^{l_{h}}\frac{M(x)}{EI_{h}}B\cdot d\left(x\right)+\\ & \int_{l_{h}}^{l_{h}+l_{b}}\frac{F_{l}\left(x\right)}{EA_{b}}\;A\cdot d\left(x\right)+\int_{l_{h}}^{l_{h}+l_{b}}\frac{M(x)}{EI_{b}}B\cdot d\left(x\right) \end{array}$$

(5) $$\begin{array}{cc} y_{output}= & 2\int_{0}^{l_{h}}\frac{F_{l}\left(x\right)}{EA_{h}}\;C\cdot d\left(y\right)+2\int_{0}^{l_{h}}\frac{M(x)}{EI_{h}}D\cdot d\left(y\right)+\\ & \int_{l_{h}}^{l_{h}+l_{b}}\frac{F_{l}\left(x\right)}{EA_{b}}\;C\cdot d\left(y\right)+\int_{l_{h}}^{l_{h}+l_{b}}\frac{M(x)}{EI_{b}}D\cdot d\left(y\right) \end{array}$$

where $A=\frac{\partial F_{l}\left(x\right)}{\partial F_{x}},B=\frac{\partial M(x)}{\partial F_{x}},C=\frac{\partial F_{l}\left(x\right)}{\partial F_{y}},D=\frac{\partial M(x)}{\partial F_{y}}$, and *E* denotes Young’s modulus of aluminum 7075. *M* is the moment, $A_{h}$ and $A_{b}$ are the cross sections of the hinge and beam; $I_{h}$ and $I_{b}$ represent the moments of inertia of the hinge and beam, respectively. In addition, these equations can be simplified as follows [35]:

(6) $$F_{l}\left(x\right)=F_{x}$$

(7) $$M\left(x\right)=\frac{1}{2}\left[F_{x}d_{h}-F_{y}\left(l_{h}+l_{b}\right)\right]$$

To further simplify, the matrix can be rewritten using $K_{11}$, $K_{12}$, $K_{21}$, and $K_{22}$ as follows:

(8) $$\left[\begin{array}{c} K_{11}\\ K_{21} \end{array}\right]=\left[\begin{array}{c} \frac{2l_{h}}{Ewt_{h}}+\frac{l_{b}}{Ewt_{b}}+\frac{6l_{h}d_{h}^{2}}{Ewt_{h}^{3}}+\frac{3l_{b}d_{h}^{2}}{Ewt_{b}^{3}}\\ \frac{6l_{h}d_{h}\left(l_{h}+l_{b}\right)}{Ewt_{h}}+\frac{3l_{b}d_{h}\left(l_{h}+l_{b}\right)}{Ewt_{b}} \end{array}\right]$$

(9) $$\left[\begin{array}{c} K_{12}\\ K_{22} \end{array}\right]=\left[\begin{array}{c} \frac{6l_{h}d_{h}\left(l_{h}+l_{b}\right)}{Ewt_{h}^{3}}+\frac{3l_{b}d_{h}\left(l_{h}+l_{b}\right)}{Ewt_{b}^{3}}\\ \frac{6l_{h}{\left(l_{h}+l_{b}\right)}^{2}}{Ewt_{h}^{3}}+\frac{3l_{b}{\left(l_{h}+l_{b}\right)}^{2}}{Ewt_{b}^{3}} \end{array}\right]$$

The relationship between $x_{input}$ and $y_{output}$ can be rewritten in a matrix form, as below:

(10) $$\left(\begin{array}{c} x_{input}\\ y_{output} \end{array}\right)=\left[\begin{array}{cc} K_{11} & K_{12}\\ K_{21} & K_{22} \end{array}\right]\left(\begin{array}{c} F_{x}\\ F_{y} \end{array}\right)$$

When the output force $F_{output}$ is assumed to be zero, the amplification ratio $R_{flexure}$ of the flexure-based mechanism is determined as:

(11) $$R_{flexure}=\frac{2y_{output}}{2x_{input}}=\frac{K_{21}}{K_{11}}$$

Further, the stiffness $K_{input}$ of the amplification mechanism can be presented as:

(12) $$K_{input}=\frac{F_{input}}{x_{input}}=\frac{2}{K_{11}}$$

Similarly, if the input force $F_{input}$ is assumed to be zero, the stiffness of the output condition is expressed in the following relationship:

(13) $$K_{output}=\frac{F_{y_{output}}}{2x_{output}}=\frac{1}{K_{22}}$$

### 2.3. Flexure-Based Parallelogram Mechanism

**Figure 7:**
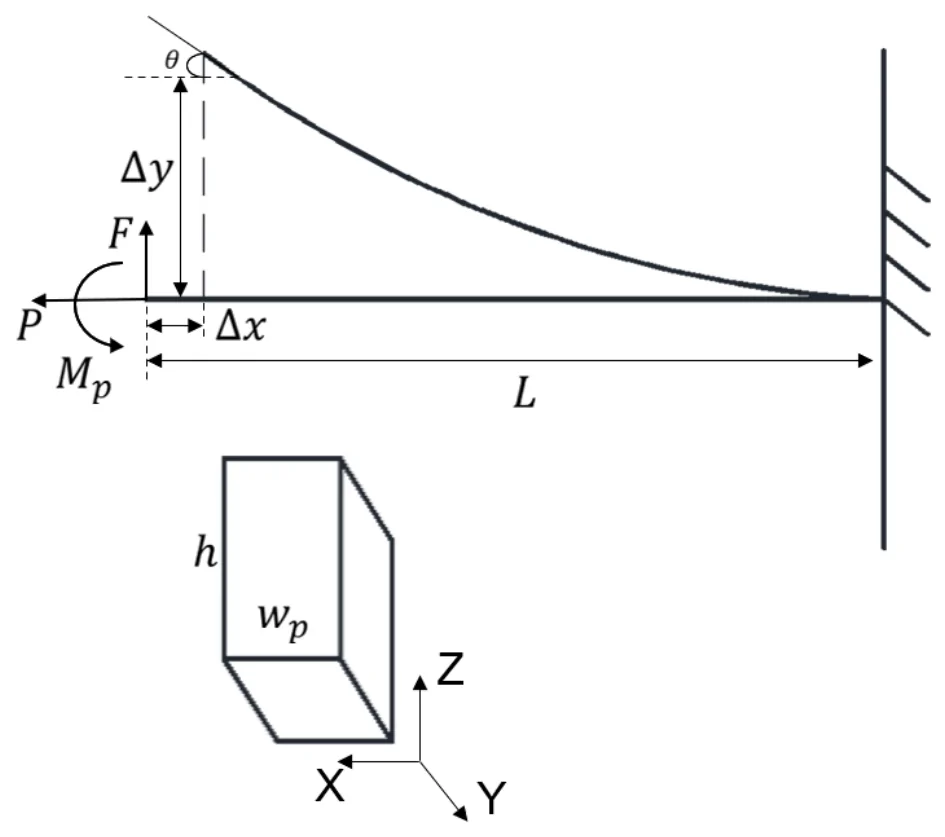
Flexure-based parallelogram beam and its cross-section.

In the proposed hybrid mechanism, parallelogram flexure beams were used at the output of the flexure-based amplification mechanism. When the deformation of a beam remains within ten percent of its length, beam constraint theory can be applied to estimate the deformation of parallelogram beams [36]. One of the parallelogram flexure structures is shown in Figure 7, and the parameters are presented in Table 2. The following equations can be developed for the parallelogram beam:

(14) $$\left[\begin{array}{c} \frac{FL^{2}}{EI}\\ \frac{M_{p}L}{EI} \end{array}\right]=\left[\begin{array}{cc} 12 & -6\\ -6 & 4 \end{array}\right]\left[\begin{array}{c} \frac{\Delta y}{L}\\ \theta \end{array}\right]+\frac{PL^{2}}{EI}\left[\begin{array}{cc} \frac{6}{5} & -\frac{1}{10}\\ -\frac{1}{10} & \frac{2}{15} \end{array}\right]\left[\begin{array}{c} \frac{\Delta y}{L}\\ \theta \end{array}\right]$$

(15) $$\begin{array}{cc} \frac{\Delta x}{L}= & \frac{\frac{PL^{2}}{EI}}{\frac{12L^{2}}{w_{p}^{2}}}+\left[\begin{array}{cc} \frac{\Delta y}{L} & \theta \end{array}\right]\left[\begin{array}{cc} -\frac{6}{10} & \frac{1}{20}\\ \frac{1}{20} & -\frac{1}{15} \end{array}\right]\left[\begin{array}{c} \frac{\Delta y}{L}\\ \theta \end{array}\right]+\\ & \frac{PL^{2}}{EI}\left[\begin{array}{cc} \frac{\Delta y}{L} & \theta \end{array}\right]\left[\begin{array}{cc} \frac{1}{700} & -\frac{1}{1400}\\ -\frac{1}{1400} & \frac{11}{6300} \end{array}\right]\left[\begin{array}{c} \frac{\Delta y}{L}\\ \theta \end{array}\right] \end{array}$$

When a parallelogram beam deforms along the Y-axis, its rotation and X-direction displacement are assumed to be zero due to the fixed supports at the other ends of the beam. The relationship between the input force and the deformation in the Y-direction can be simplified from Equations (14) and (15) as follows:

(16) $$\frac{PL^{2}}{EI}=\frac{\frac{6}{10}\left({\frac{\Delta y}{L}}^{2}\right)}{\frac{w_{p}^{2}}{12L^{2}}+\frac{\Delta y^{2}}{700L^{2}}}$$

(17) $$\frac{FL^{2}}{EI}=\frac{\Delta y}{L}\left(12+\frac{6PL^{2}}{5EI}\right)$$

For four parallel flexure beams, the input force needs to be increased four times to obtain the same output displacement. Therefore, Equation (17) can be modified as:

(18) $$\frac{FL^{2}}{EI}=4\frac{\Delta y}{L}\left(12+\frac{6PL^{2}}{5EI}\right)$$

The stiffness of the parallelogram beams is calculated as:

(19) $$K_{para}=\frac{F}{\Delta y}=4\frac{EI}{L^{3}}\left(12+\frac{6PL^{2}}{5EI}\right)$$

### 2.4. Stiffness Characterization for Amplification Mechanism

The four parallelogram elements are configured in parallel with the motion of the flexure-based structure, allowing them to function as elastic elements. The following equations express the relationship between the elastic load $F_{elastic}$ and elastic stiffness $K_{elastic}$, respectively.

(20) $$F_{elastic}=K_{elastic}d_{hybrid}$$

where $d_{hybrid}$ is the output of hybrid mechanism. The elastic force acts opposite to the input forces that activate the amplification mechanism. Therefore, the total input and output motions of the hybrid mechanism are developed as:

(21) $$X_{input}=X_{in_{bridge}}-X_{in_{elastic}}$$

(22) $$Y_{output}=2\left(Y_{out_{bridge}}-Y_{out_{elastic}}\right)$$

where $X_{in_{bridge}}$, $Y_{out_{bridge}}$, and $X_{in_{elastic}}$ and $Y_{out_{elastic}}$ are the input and output of the bridge-type structure, and the elastic input and output displacements of the parallelogram mechanism, respectively. These deformations can be presented as follows:

(23) $$Y_{out_{bridge}}=X_{in_{bridge}}R_{flexure}=\frac{F_{x_{input}}}{K_{input}}R_{flexure}$$

(24) $$X_{in_{elastic}}=\frac{Y_{out_{elastic}}}{R_{flexure}}=\frac{F_{elastic}}{2K_{output}R_{flexure}}$$

Finally, the following equations describe the input force and the input and output stiffnesses of the combined hybrid mechanism:

(25) $$F_{x_{input}}=K_{input}^{hybrid}\frac{Y_{output}}{2R_{flexure}}$$

(26) $$K_{input}^{hybrid}=\frac{K_{input}\left(K_{output}+K_{elastic}\right)}{K_{output}}$$

(27) $$K_{output}^{hybrid}=\left(K_{output}+K_{elastic}\right)$$

where $F_{x_{input}}$ is the input force, $K_{input}^{hybrid}$ and $K_{output}^{hybrid}$ are the input and output stiffnesses of the combined hybrid mechanism, respectively.

### 2.5. Parameter Identification and Analysis for Long-Motion Stage

**Figure 8:**
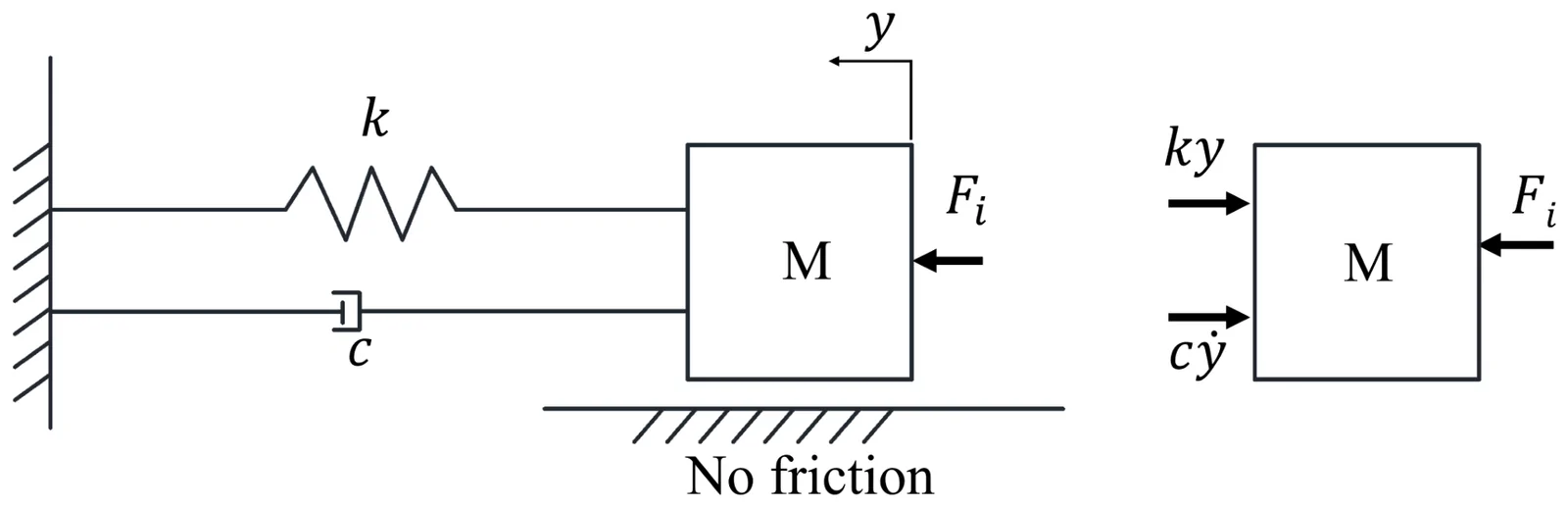
Free body diagram of long-range motion.

The entire frame of the hybrid mechanism is positioned on a sliding mechanism (slider block), which provides the friction-free movement of the long-range motion stage. Figure 8 presents the free body diagram of the long-range motion stage, where *m* = 0.37207 kg is the mass of the entire mechanism positioned on the slider blocks. *F* is the input force, $\ddot{y}$, $\dot{y}$ and *y* are acceleration, velocity, and displacement of the mechanism, respectively; *k* = 8009 N/m is the stiffness and *c* = 3.2459 Ns/m is the damping coefficient of the long-range motion stage. Therefore, the following equations can be written to present the dominant parameters:

The dynamic equation is of second order and can be expressed as:

(28) $$m\ddot{y}+c\dot{y}+ky=F$$

The undamped natural frequency $\omega_{n}$ is found by setting damping c=0.

(29) $$\omega_{n}=\sqrt{\frac{k}{m}}$$

The damping ratio can be obtained as $\epsilon =\frac{c}{2\sqrt{km}}$, and then the damped natural frequency $\omega_{d}$ can be expressed as follows:

(30) $$\omega_{d}=\omega_{n}\sqrt{1-\epsilon^{2}}$$

The resonant frequency of the long-range motion stage was determined as 23.35 Hz, which is 7.4% lower than the value of 25.21 Hz obtained from the results of the finite element analysis reported in Section 3.2.

## 3. Computational Analysis

The characteristics of the hybrid mechanism were analyzed using computational software (ANSYS 2022 R1). Aluminum alloy 7075 was chosen for the manufacturing of this hybrid structure, with the material properties shown in Table 3.

### 3.1. Static Analysis

**Figure 9:**
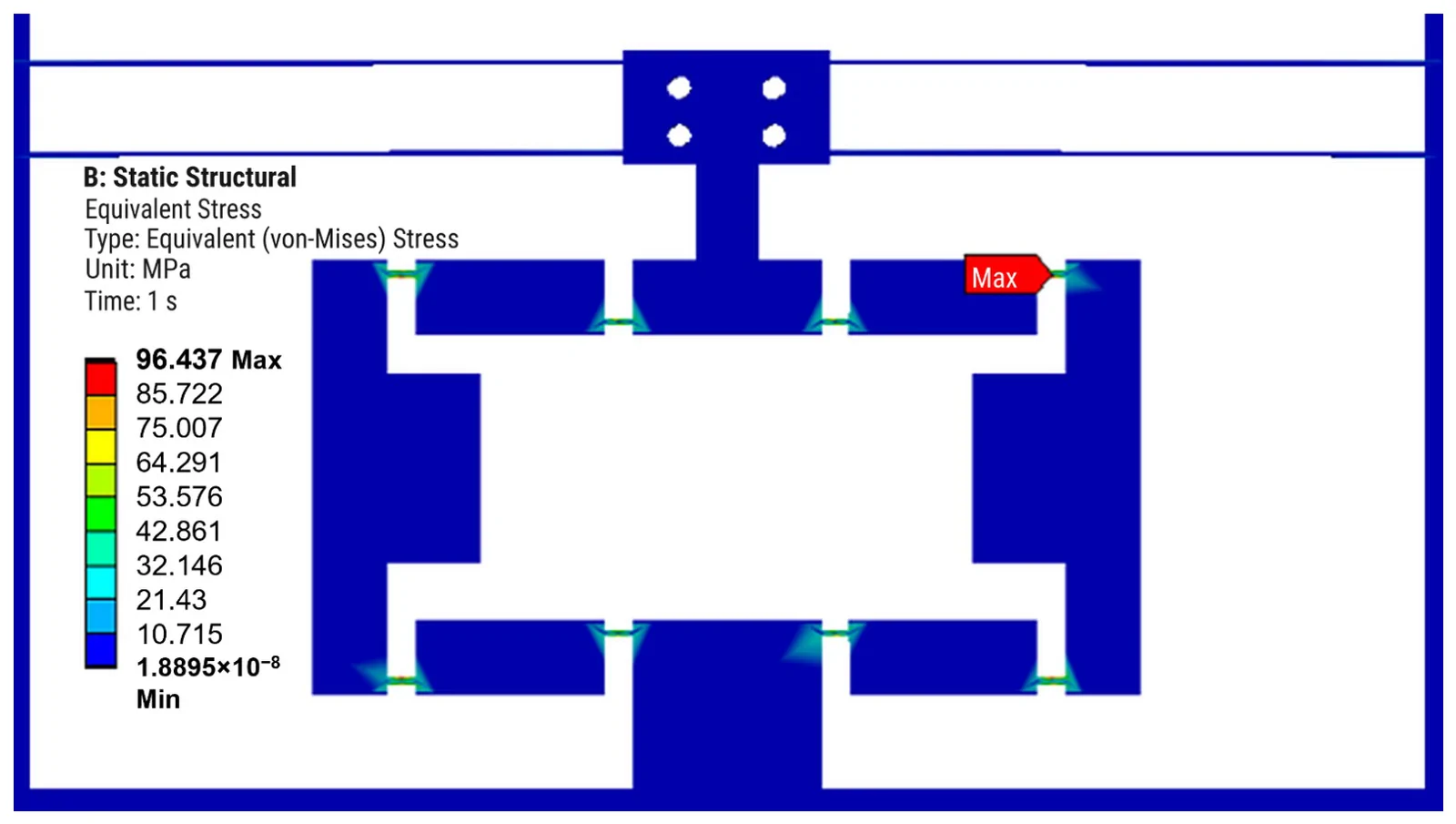
Equivalent stress of the amplification mechanism.

ANSYS Workbench was used to perform the computational analysis of the hybrid mechanism to determine its maximum stress and total deformation. The maximum input displacement was set to 40 μm, corresponding to the full stroke of the piezoelectric actuator. The maximum equivalent stress was captured at the hinge $H_{4}$, reaching 96.40 MPa, which is well below the 503 MPa yield strength of aluminum 7075, and corresponds to a realized safety factor of 5.2, as shown in Figure 9. The result demonstrates that the proposed hybrid mechanism can function effectively under the specified input condition. Furthermore, in this flexure-based mechanism, the input stiffness and output stiffness were obtained by applying a force of 10 N to the input ends of amplification mechanism and the output platform. The input and output stiffnesses were thereby identified as 2325.58 N/mm and 61.15 N/mm, respectively.

### 3.2. Dynamic Analysis

**Figure 10:**
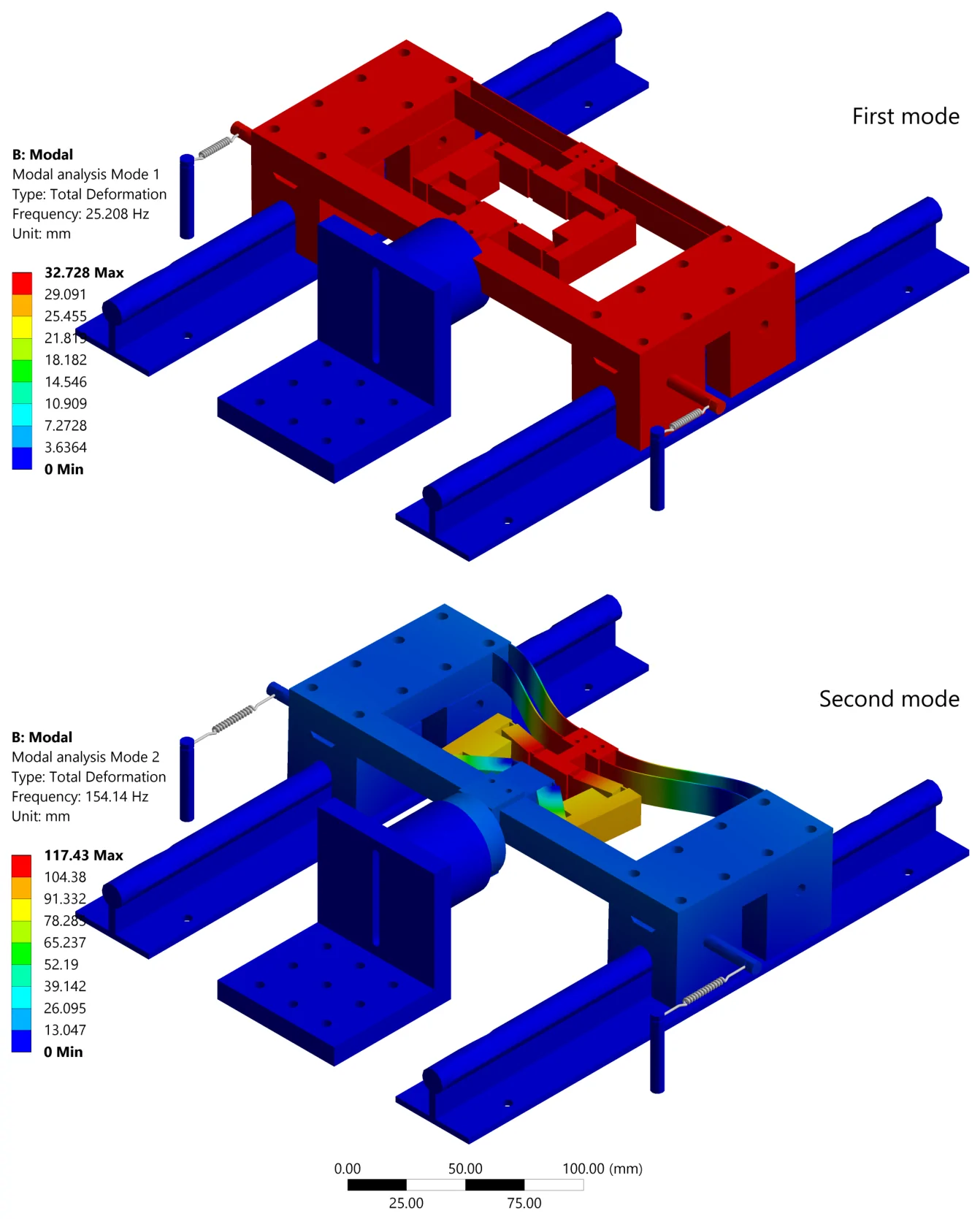
First two resonant modes of the hybrid mechanism.

The dynamic analysis of the hybrid mechanism was conducted to gain knowledge of its natural frequencies. The first two modes of the structure are presented in Figure 10. The first resonant frequency was determined to be 25.21 Hz, corresponding to the long-range motion provided by the voice coil motor. The second resonant frequency was found to be 154.14 Hz, corresponding to the motion of the second stage of this hybrid mechanism when the piezoelectric actuator is activated. The precision motion stage can therefore be driven at a much higher frequency than the long-range motion stage actuated by the voice coil motor. The remaining modes correspond to unwanted motions of the mechanism and are unlikely to be activated in practice.

## 4. Experiment

### 4.1. System Identification (SI)

**Figure 11:**
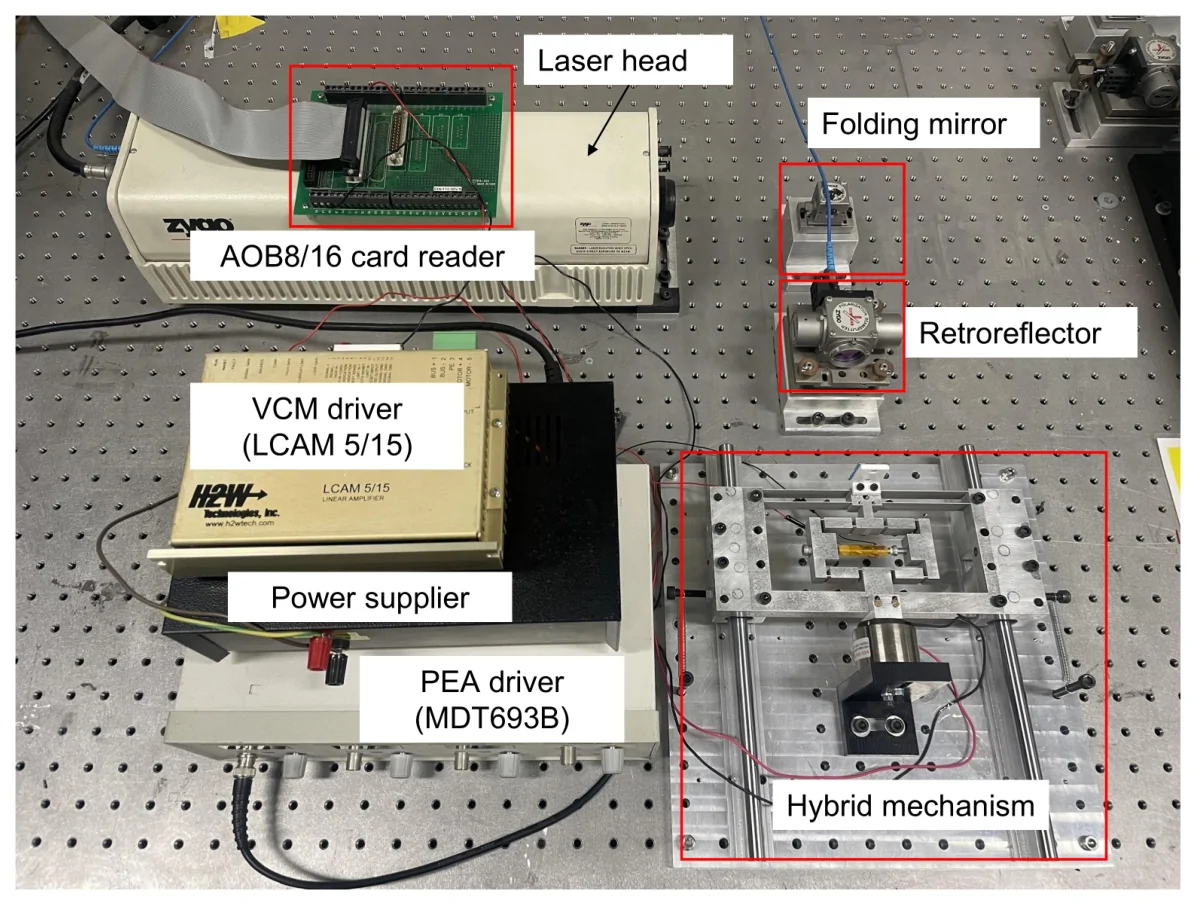
Experimental setup for system identification.

**Figure 12:**
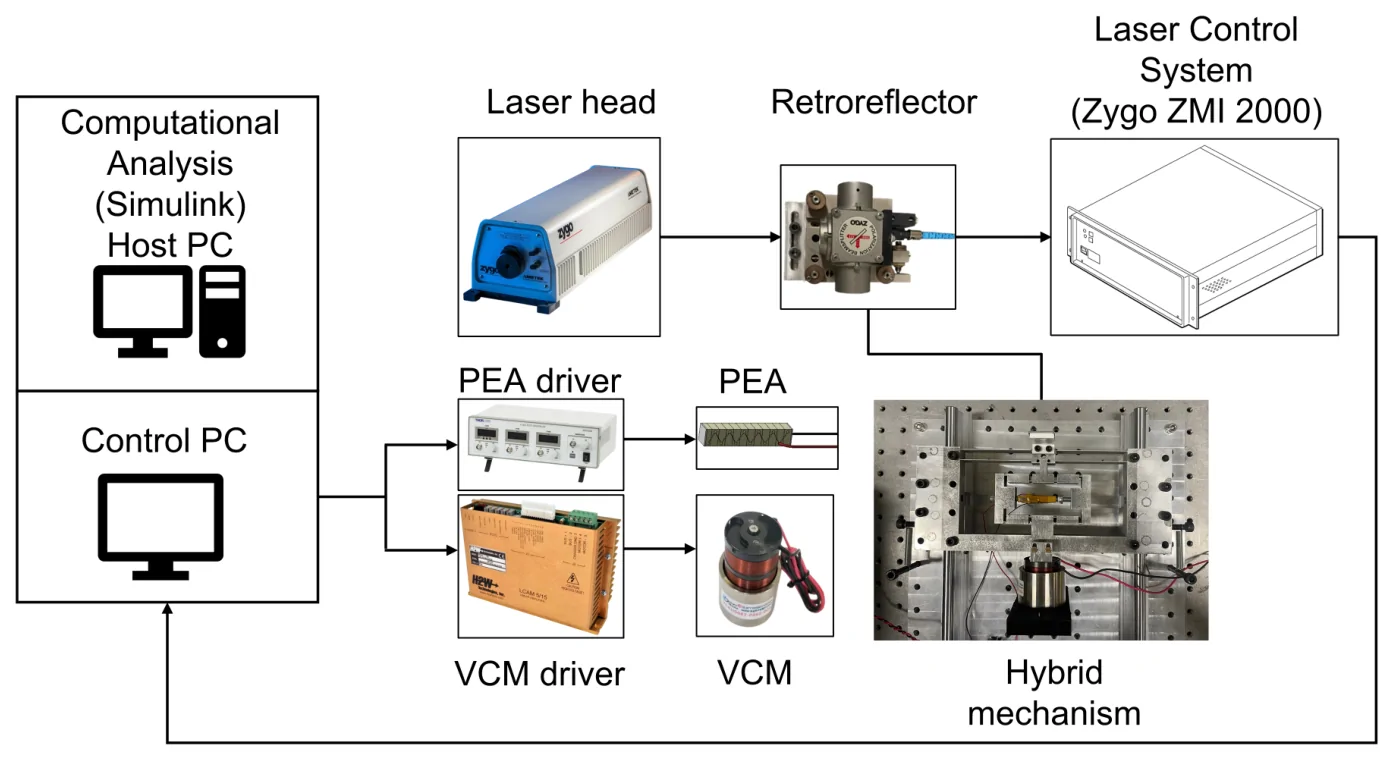
Sensing and control architecture for experimental system identification.

Figure 11 and Figure 12 show the experimental setup and system architecture, respectively, for the system identification. The VCM (model VCAR0087-0062-00A, SUPT Motion, Suzhou, China) and the piezoelectric actuator (model PK4GYP2, Thorlabs, Newton, NJ, USA) were connected to the LCAM 5/15 driver (i.e., VCM amplifier from H2W Technologies, Santa Clarita, CA, USA), and the piezoelectric driver (Thorlabs, Newton, NJ, USA), respectively. MATLAB/Simulink R2022a was used to transfer the control signals from the host PC to the target PC, where an AOB8/16 analog I/O board relayed the signals from the target PC to the actuator drivers. The VCM driver connected to the coil of the VCM, was responsible for articulating the VCM and thus driving the long-range motion stage, to enable long-range motion, while the piezoelectric driver was connected to activate and control the precision motion stage. The motion sensing and measurement was conducted by a Zygo laser interferometric sensing and measurement system (Zygo Corporation, Middlefield, CT, USA; resolution 0.31 nm), with a reflector placed on the mechanism positioning platform (i.e., end-effector) to measure the displacement of the hybrid mechanism.

(31) $$G_{s}=\frac{1.867e^{6}}{S^{4}+78S^{3}+8.975e^{5}S^{2}+4.143e^{7}S+2.233e^{10}}$$

**Figure 13:**
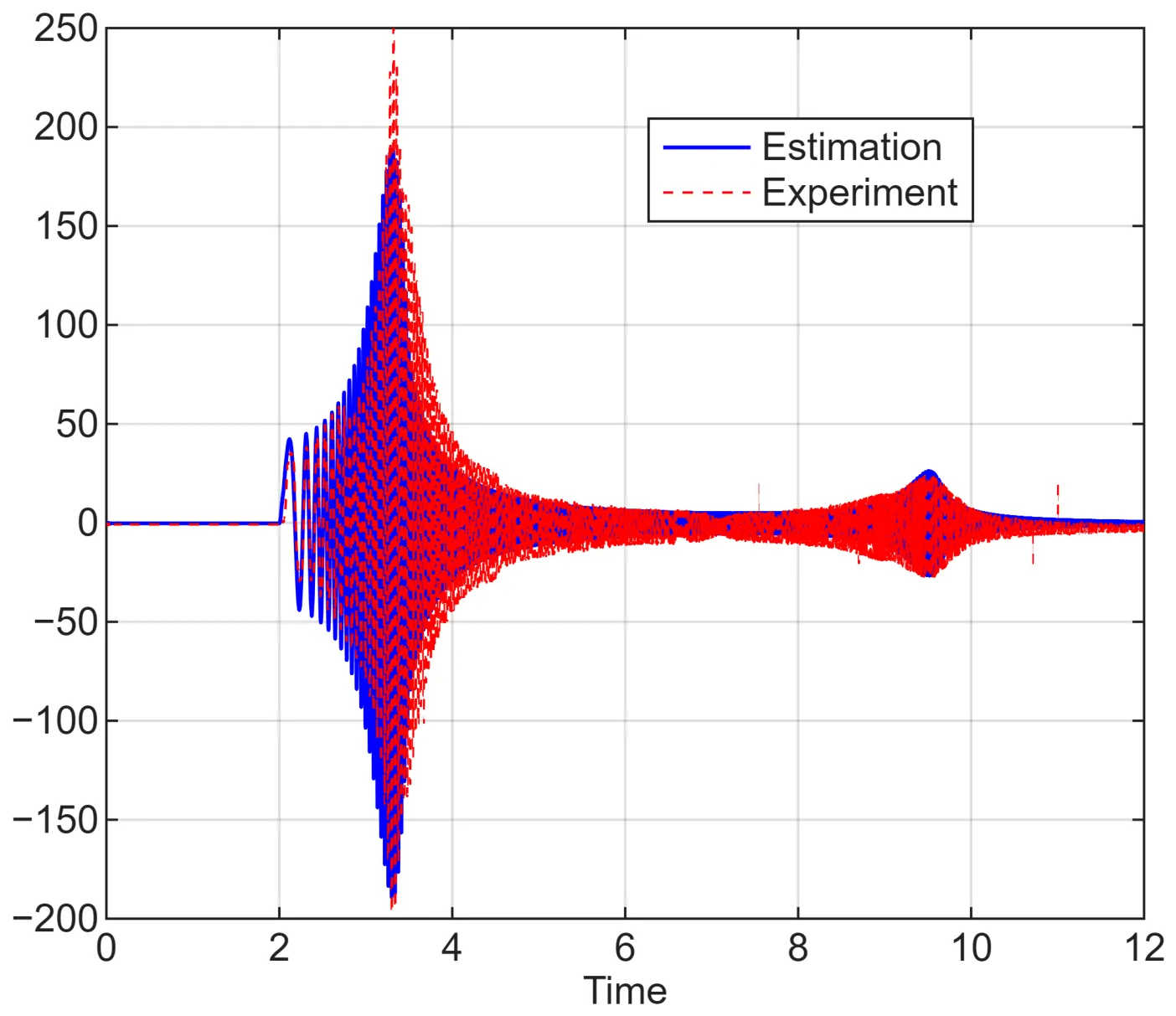
Estimated time response.

**Figure 14:**
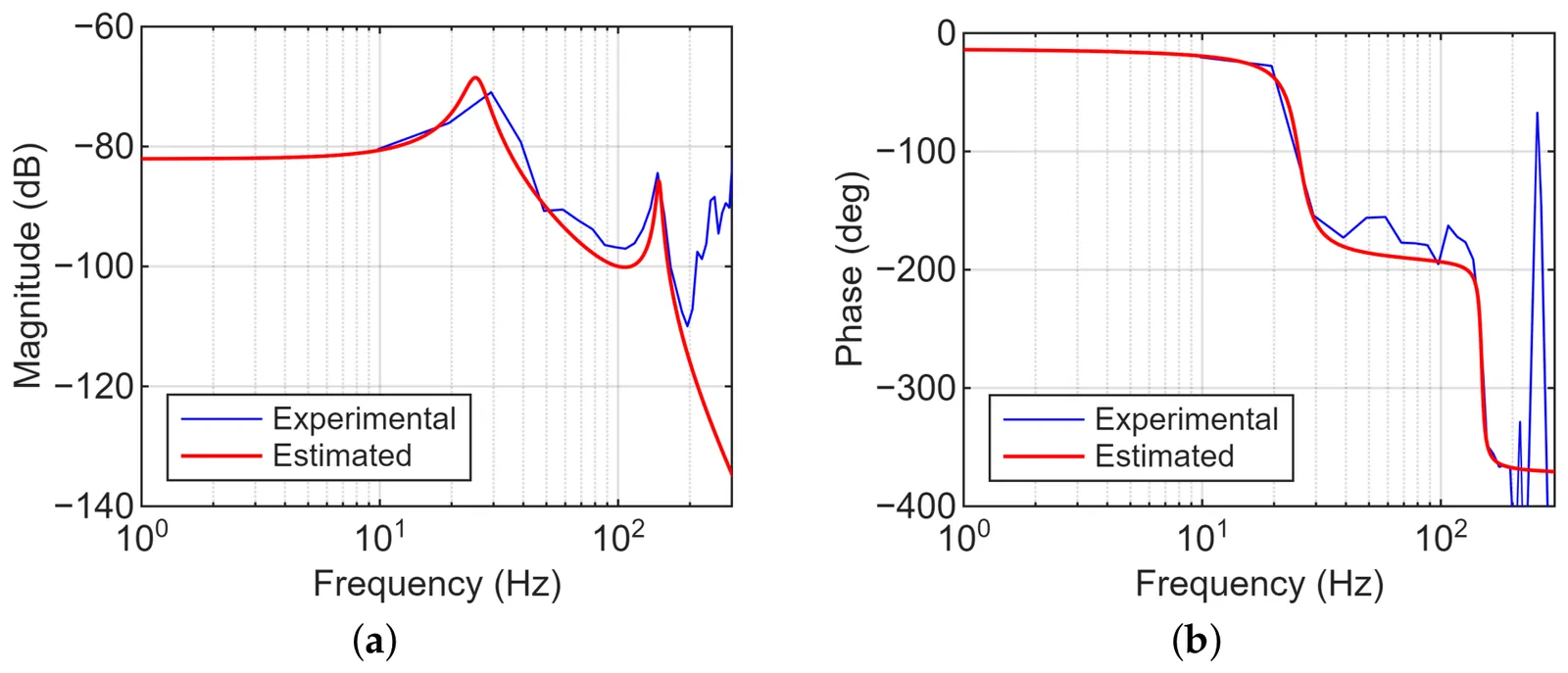
Frequency analysis: (**a**) magnitude Bode plot; (**b**) phase angle Bode plot.

The transfer functions of this hybrid flexure-based mechanism were identified using system identification in MATLAB/Simulink, and the resonant frequencies were then compared with those obtained from the analytical model. In order to identify the transfer functions, a swept-sine signal from 0.1 Hz to 300 Hz was applied, with 30,000 measurement points. As a result, the output displacement of the entire hybrid motion stage was measured, and an output model was estimated to identify the transfer function of the proposed hybrid mechanism, as shown in Figure 13. Therefore, a fourth-order transfer function was identified, as defined in Equation (31). The frequency response plots of the mechanism were presented in Figure 14a,b. The resonant frequencies of this hybrid mechanism are identified to range between 25 Hz and 148 Hz, corresponding to the long-range and precisionC motion stages, respectively.

### 4.2. Hysteresis Analysis for Open-Loop Control

**Figure 15:**
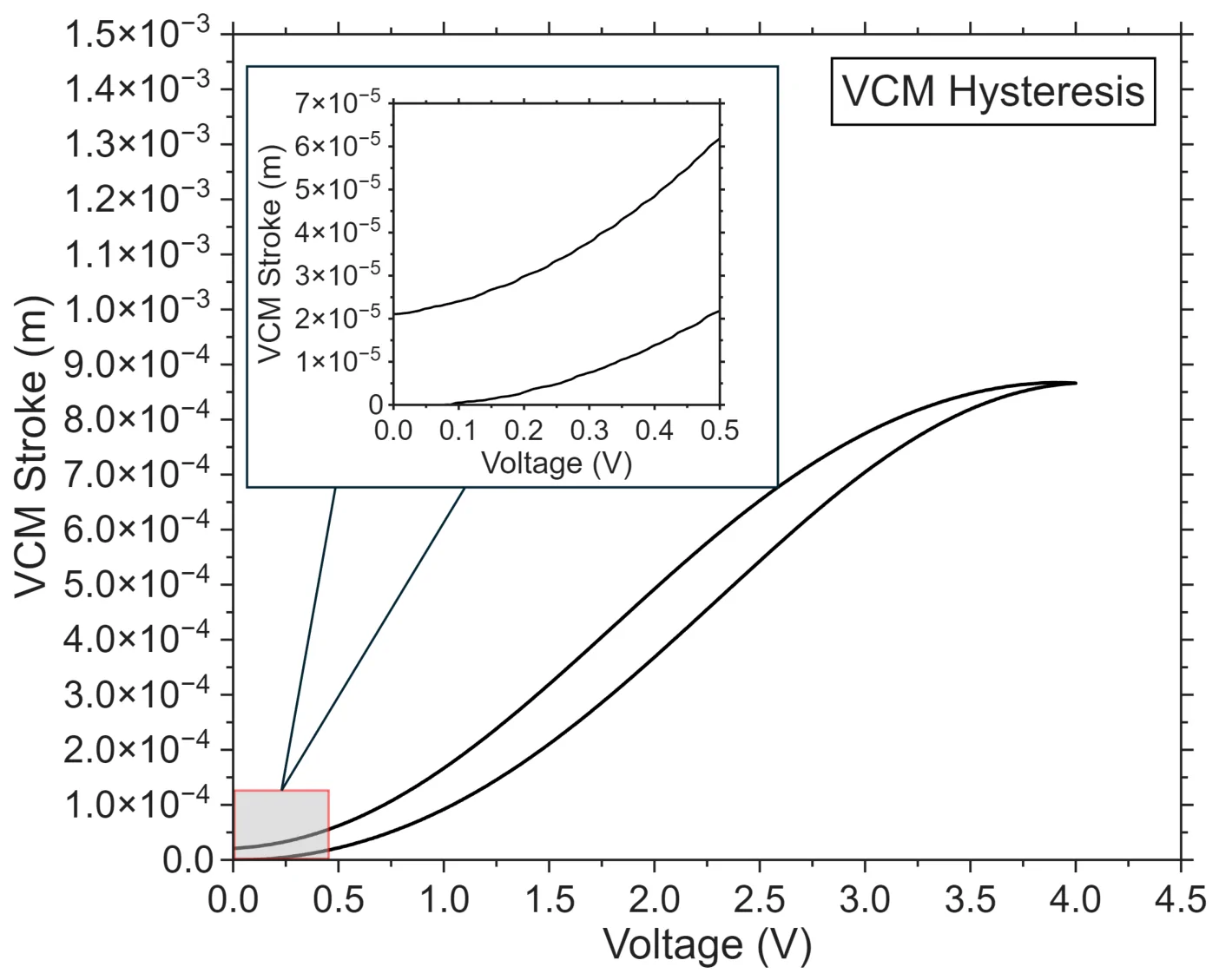
Hysteresis of the long-range motion stage of the hybrid mechanism.

**Figure 16:**
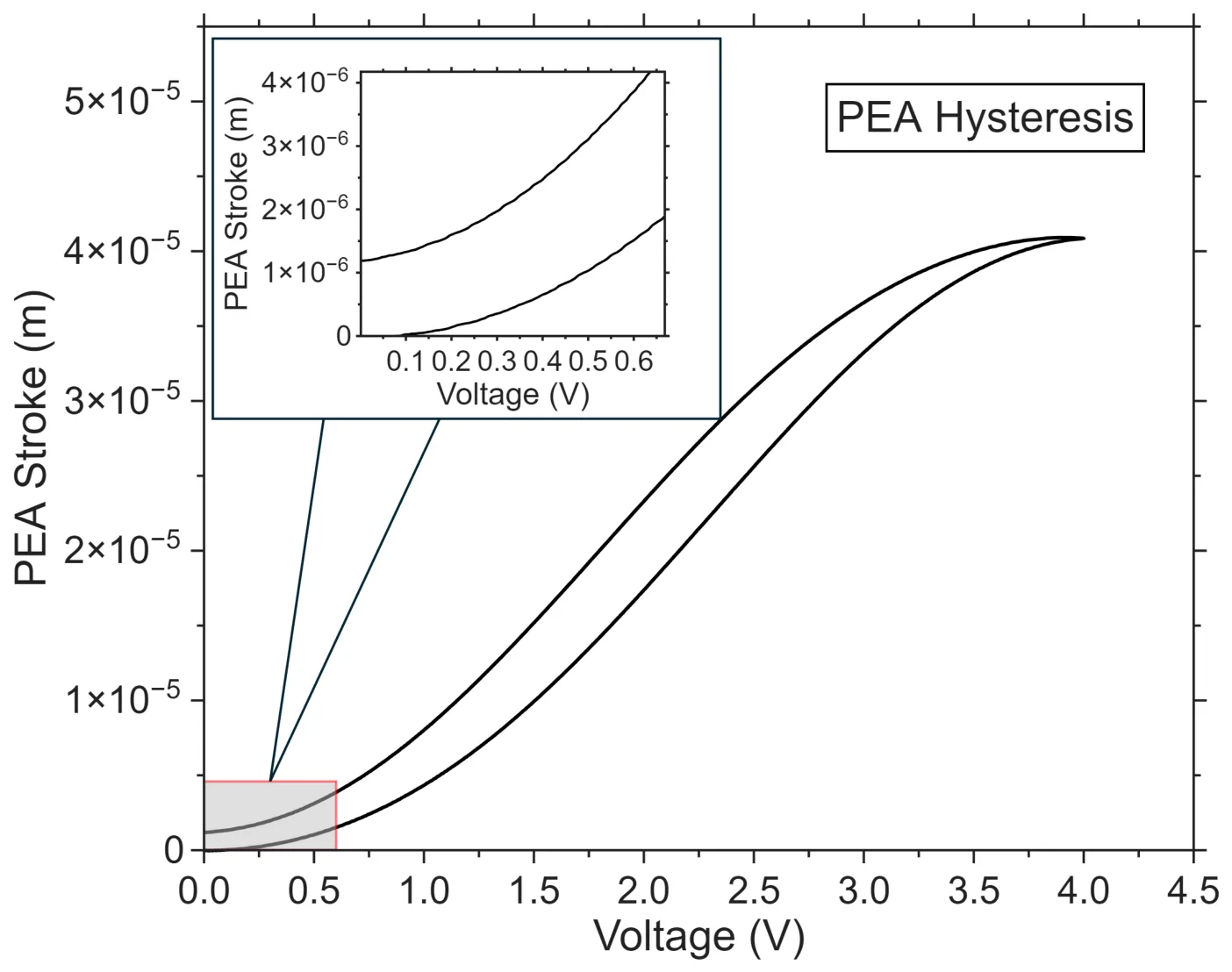
Hysteresis of the precision motion stage of the hybrid mechanism.

For open-loop control, experiments were conducted to establish the effects of hysteresis for the long-range and precision motion stages. These experiments were performed using a sine signal of 4 volts under a frequency of 1 Hz. Since this hybrid mechanism is designed to operate only in the positive direction, and hence only positive signals were applied to the voice coil motor and piezoelectric actuator. Figure 15 and Figure 16 illustrate the hysteresis of the long-range motion and precision motion stages, respectively. The hysteresis of the long-range motion stage is approximately 20 μm, while the precision motion stage, driven by the piezoelectric actuator, exhibits a hysteresis of about 1.20 μm. Because a voice coil motor is a Lorentz-force actuator and is inherently free of magnetic hysteresis, the loop observed in the long-range stage is attributed principally to friction and stiction in the linear bearing blocks (i.e., sliding mechanism) and to the return springs, rather than to the actuator itself.

### 4.3. Hybrid Mechanism Control Architecture

**Figure 17:**
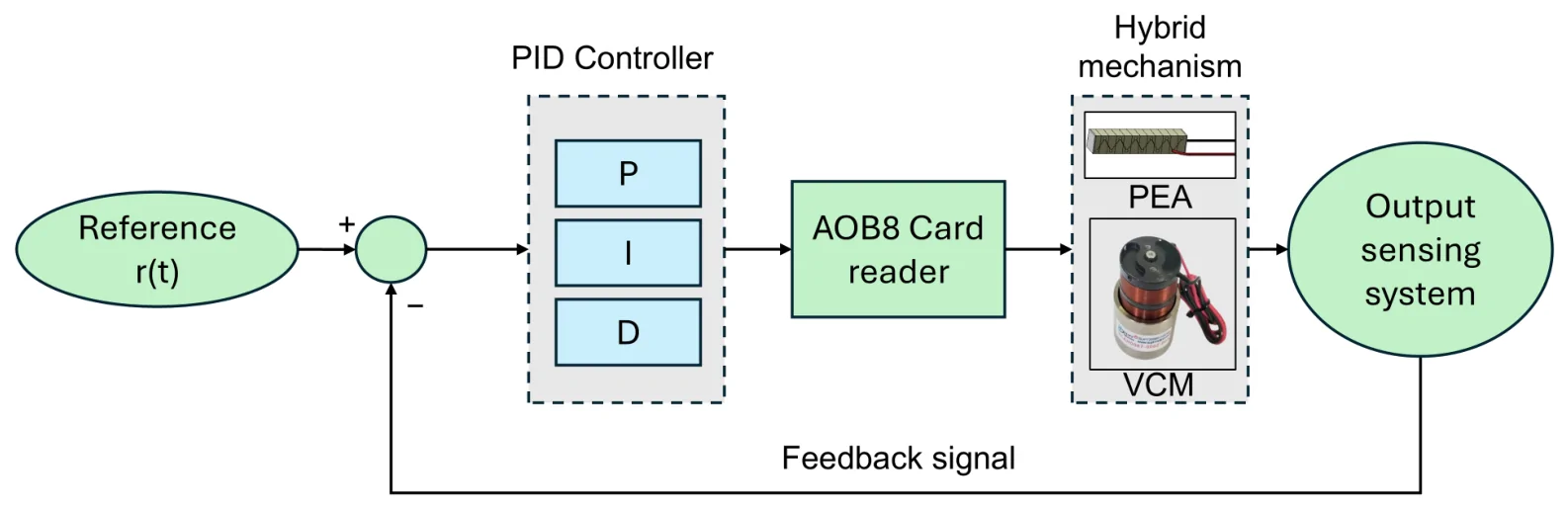
System architecture using PID control scheme.

**Figure 18:**
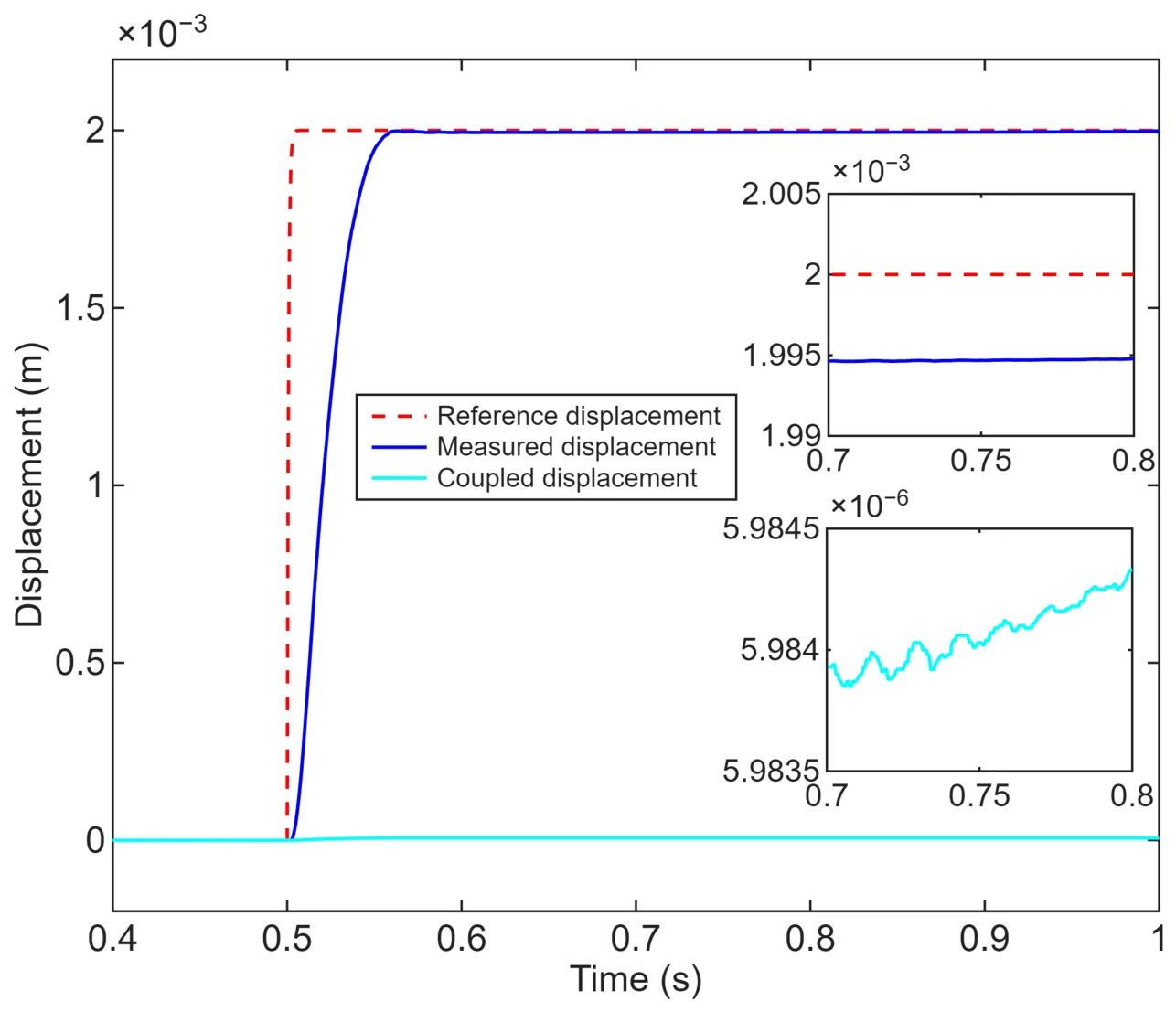
Long-range motion control: Step input.

**Figure 19:**
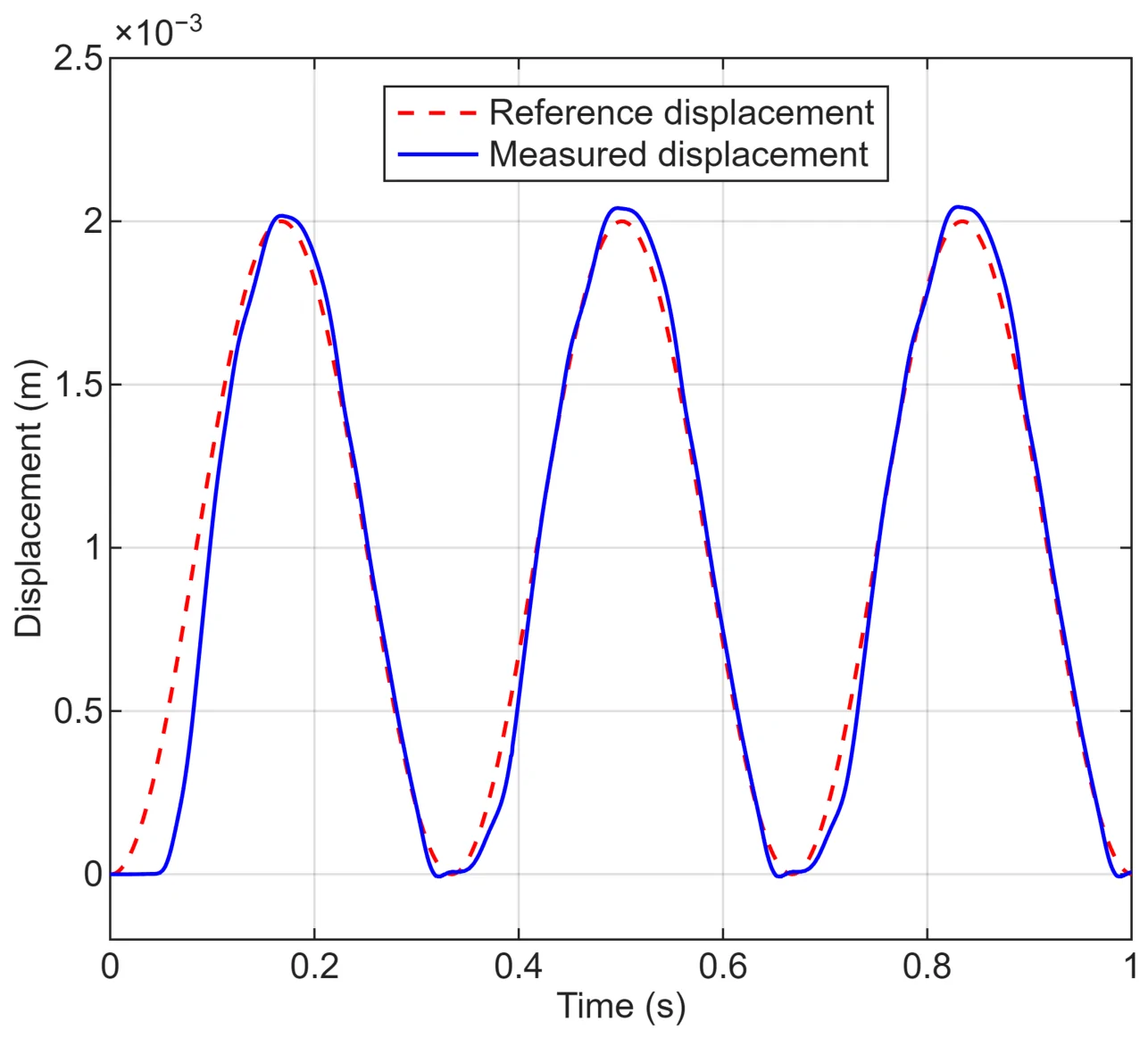
Long-range motion control: Sine input.

A Proportional-Integral-Derivative (PID) controller was implemented to generate the motions of the proposed hybrid mechanism, as shown in Figure 17. Both long-range and precision motion stages were tested under step and sinusoidal input signals to evaluate their dynamic response and the coupling displacement. The long-range motion stage was initially actuated by the VCM alone under both input types. Throughout this work, the coupling displacement is defined as the ratio of the measured off-axis displacement to the commanded on-axis displacement. Under both step and sinusoidal excitations, the motion stage achieved a maximum displacement of approximately 2 mm. For the step input, the measured output closely tracked the reference signal, demonstrating excellent tracking accuracy, and the coupling displacement was measured at about 0.3%, as shown in Figure 18. However, in the case of the sinusoidal input, an initial deviation between the reference and measured signals was observed at the beginning of the control cycle, as shown in Figure 19. This transient error was primarily attributed to inertial effects arising from the spring elements and their mounting configuration.

**Figure 20:**
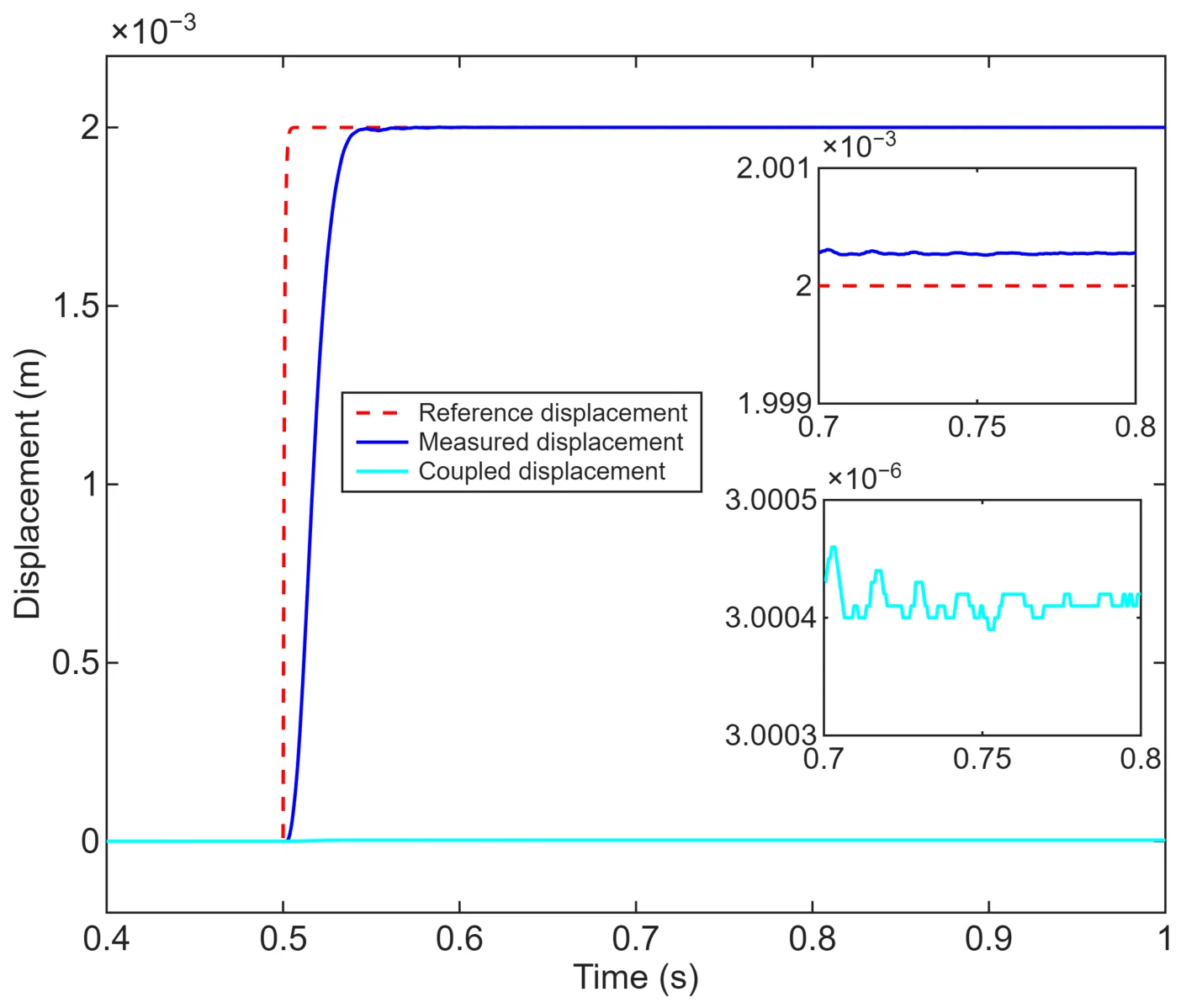
Hybrid motion control for Step input.

**Figure 21:**
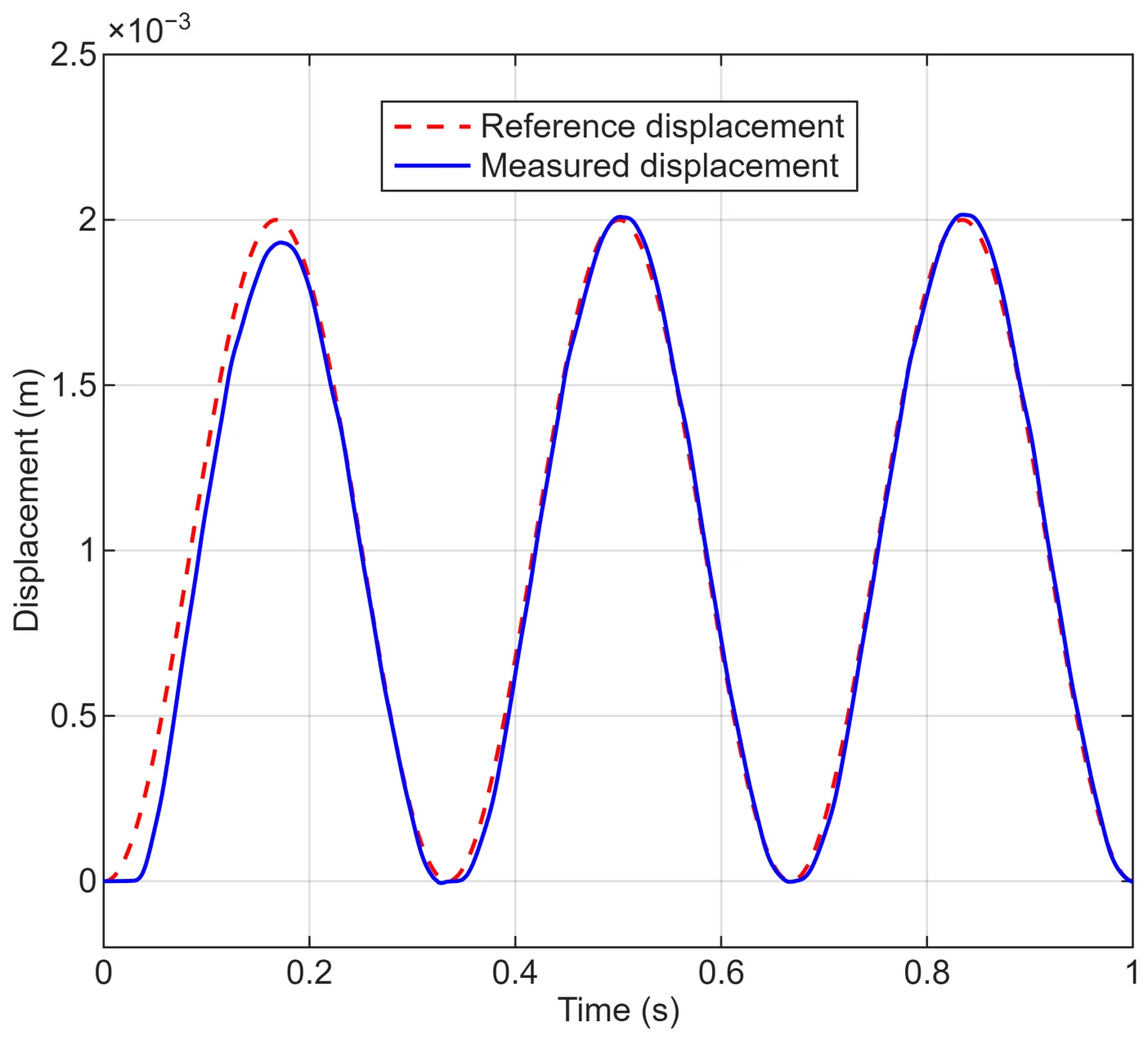
Hybrid motion control for Sine input.

Similarly, the combined hybrid motion stage was tested using step and sinusoidal input signals, with displacement provided by both VCM and PEA. The stage exhibited satisfactory tracking performance under the step input, accurately following the reference signal with the coupling motion recorded at 0.15%, which demonstrates the effectiveness of decoupling parallelogram beam mechanism (parallelogram beams) in the proposed hybrid mechanism, as shown in Figure 20. Under sinusoidal excitation, the response tracked the reference more closely than with VCM actuation alone, as illustrated in Figure 21. Therefore, the hybrid mechanism achieved both a lower tracking error and less coupled motion than the VCM-only configuration. Nevertheless, some residual errors remained at the start of each test and on return to the initial position, caused by the return springs.

**Figure 22:**
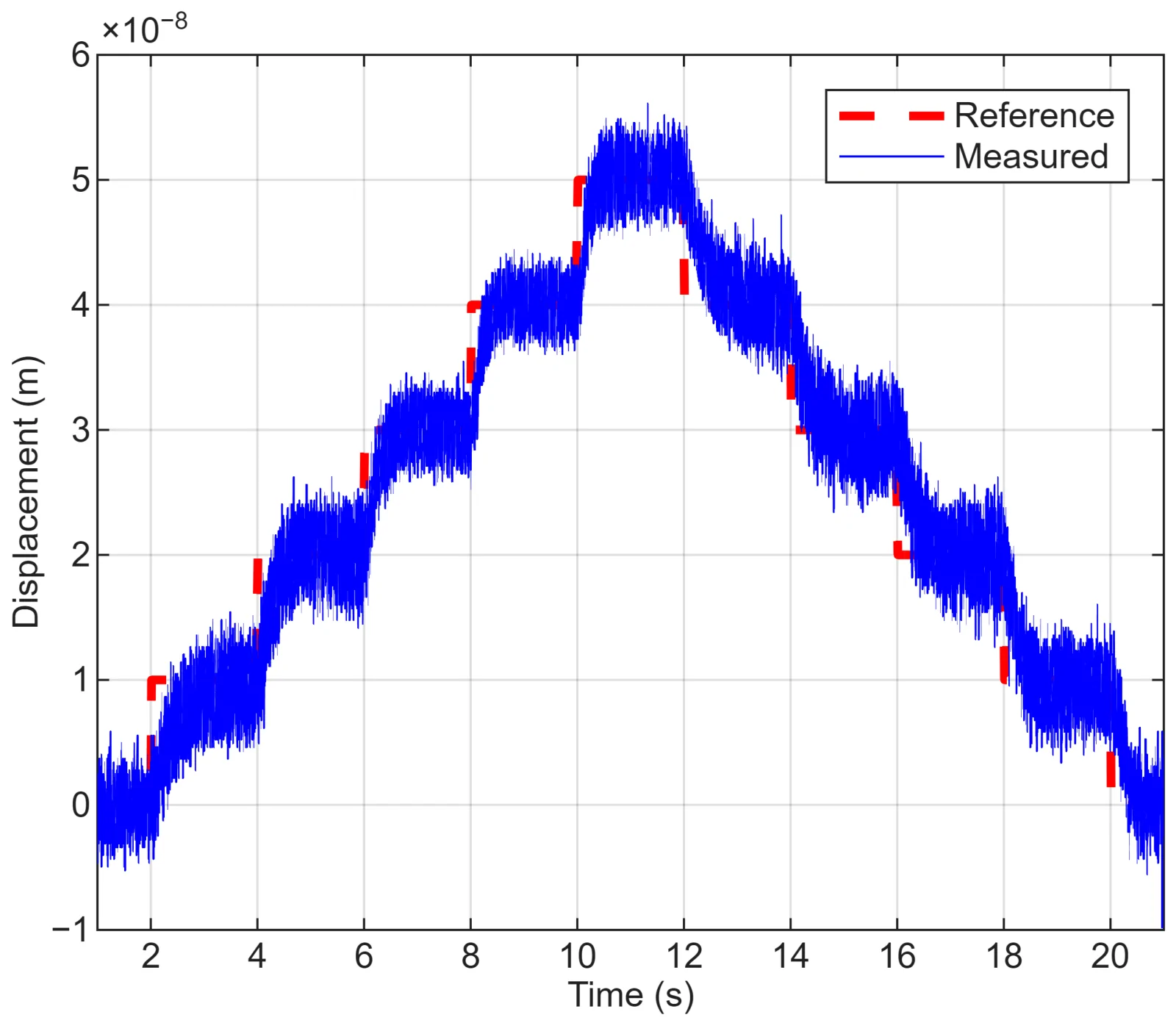
Experimental results for the resolution of the long-range motion stage.

**Figure 23:**
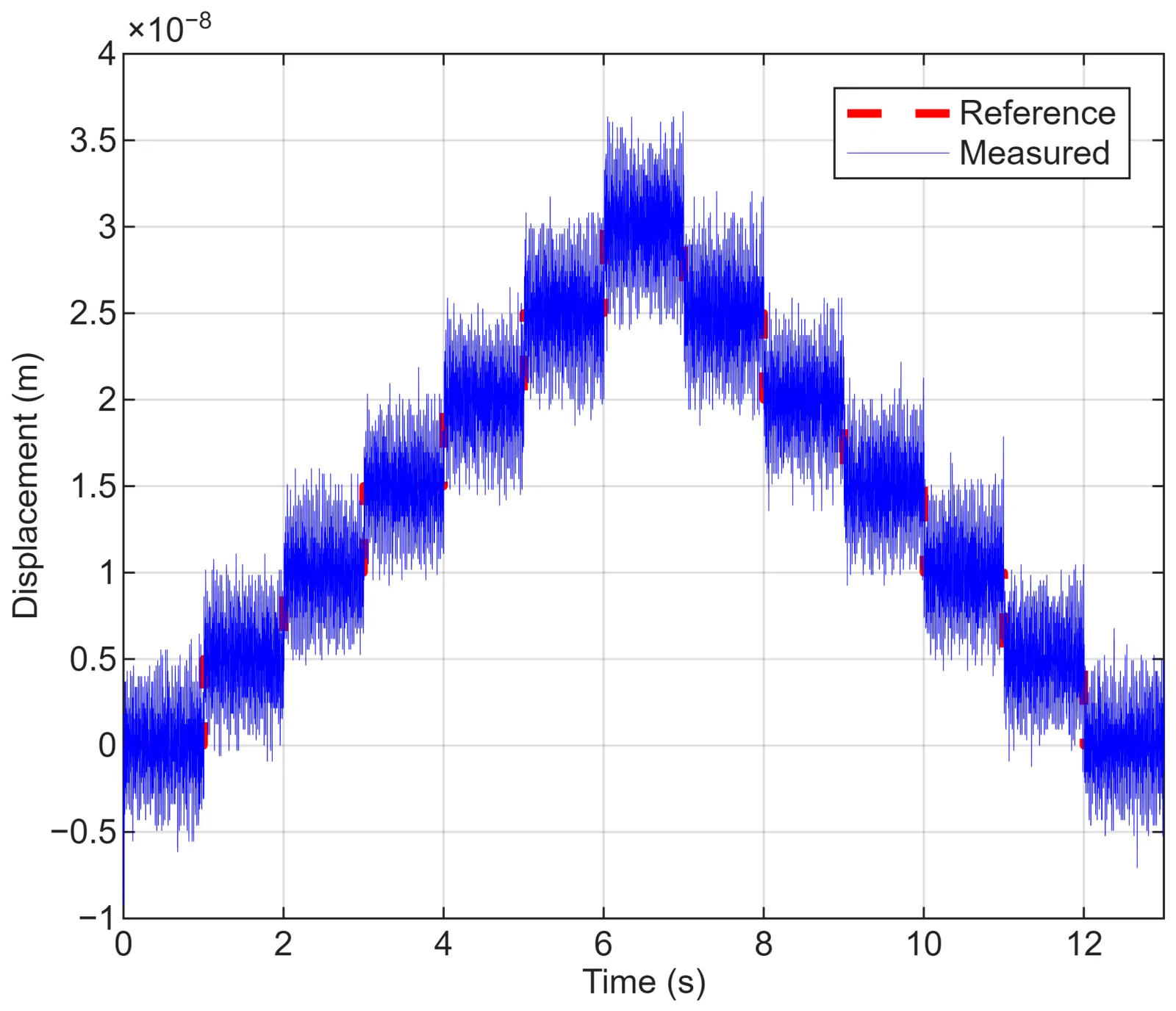
Experimental results for the resolution of the hybrid motion stage.

To further evaluate the accuracy of each stage within the hybrid mechanism, a stair-step input signal was applied to assess the mechanism’s resolution. The results revealed that the long-range stage achieved a resolution of approximately 10 nm, while the precision stage reached around 5 nm, as shown in Figure 22 and Figure 23. These results confirm the resolution of both motion stages and validate the measurement approach adopted for the hybrid system.

### 4.4. Experimental Analysis and Discussion

In the amplification mechanism, the identified amplification ratio is 4.38, which is 3.42% higher than the value obtained by FEA. The calculated input and output stiffnesses of the mechanism are 2231.81 N/mm and 60.18 N/mm, respectively. The above results differ by 4.12% and 1.60% from the results obtained using computational analysis technique, as shown in Table 4. The results of the system identification illustrate that the resonant frequencies of long-range and precision motion stages are 25 Hz and 148 Hz, respectively. These are 0.84% and 4.14% lower than the frequencies obtained from finite element analysis, as shown in Table 5. Furthermore, for the open-loop control scheme, the long-range motion stage has a hysteresis of about 20 μm, which is much higher than the hysteresis level of the precision motion stage at 1.20 μm.

The experimental results confirm that the proposed hybrid motion system achieves the intended amplification and motion performance. The relatively small discrepancies indicate reasonable agreement between the analytical/computational models and the physical mechanism, while likely reflecting manufacturing tolerances, assembly conditions, and measurement uncertainty. The close agreement supports the validity of the finite-element model for estimating the stages’ dynamic behavior. The higher resonant frequency of the precision stage also indicates its greater suitability for rapid, micropositioning motion. Furthermore, the substantial difference of hysteresis between long-range motion stage and precision motion stage highlights the limitations of relying on the long-range actuator alone for accurate positioning and motivates the use of the precision stage to compensate for nonlinearities and improve the precision motion control.

For the closed-loop control scheme, the hybrid system reduces the rise time from 32.40 ms to 18.90 ms, and the settling time from 51.90 ms to 32.10 ms, as shown in Table 6. Additionally, the overshoot is greatly minimized from 24.65% to 5.70%, indicating enhanced stability and responsiveness. The experimental results demonstrate that the hybrid motion improves dynamic performance with the recorded coupled displacement of 0.15%, compared to 0.3% obtained from the single long-range motion stage.

The improved transient response is attributed to the coordinated operation of the VCM and PEA stages. The VCM provides rapid long-range motion, while the PEA compensates for residual positioning error with fine motion. The decoupling flexure bridge and bearing slider blocks reduce mechanical interference and parasitic displacement between the stages, allowing the PEA to perform precision correction without being significantly disturbed by VCM motion. This coordinated actuation supports improved positioning accuracy and stability.

**Figure 24:**
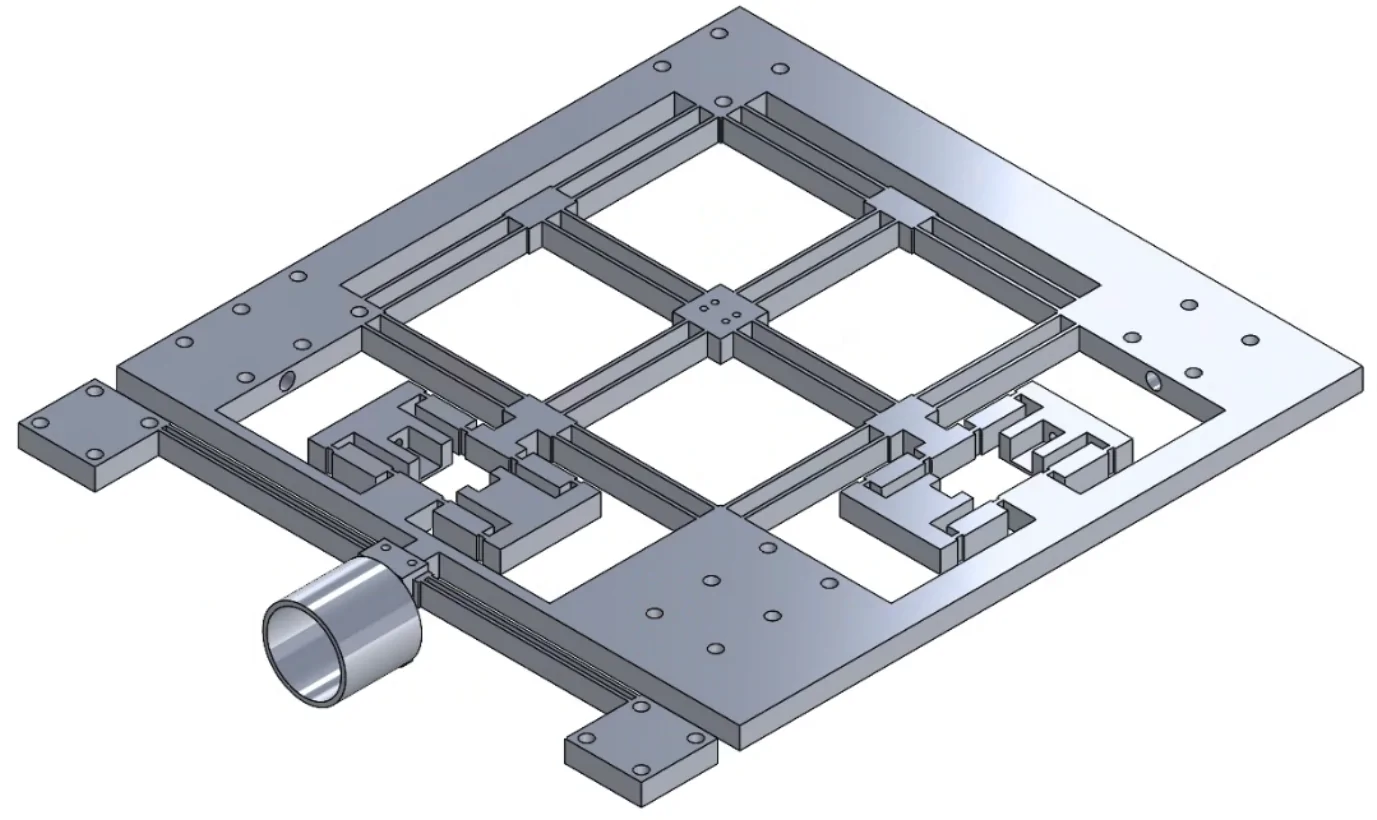
Two-degree-of-freedom hybrid mechanism.

Errors during the initial and return phases remain a limitation of the present design. A two-degree-of-freedom hybrid mechanism is therefore proposed for future work, in which the PEA is enclosed in a housing to eliminate mounting error and the return springs are replaced by four parallelogram beams, as shown in Figure 24.

The performance of this hybrid mechanism was evaluated against other long-range mechanisms, focusing on travel range and resolution. Table 7 compares the reported performance of the proposed mechanism with that of the stages in [6,28]. The proposed hybrid mechanism achieves a larger travel range (2.00 mm) and finer resolution (5 nm). Earlier hybrid designs were reported to experience significant interference between their stages, causing deformation of the precision stage during long-range motion. In contrast, the proposed design integrates the VCM long-range stage and PEA precision stage with decoupling mechanisms to minimize interstage interference. This enables extended travel while preserving precision-stage performance and achieving high-resolution positioning.

## 5. Conclusions

This paper presented a flexure-based hybrid mechanism that uses a VCM for long-range motion and a PEA for high-resolution positioning. The performance of the mechanism was analyzed using both the analytical model and finite element analysis, and verified experimentally. The amplification ratio and the input and output stiffnesses differ by no more than 4.12% between the two methods, indicating that both provide a reliable representation of the stiffness characteristics of the system. The resonant frequencies obtained from the finite element analysis and from experimental system identification differ by no more than 0.84% for the long-range stage and 4.14% for the precision motion stage. For the closed-loop control, the experimental evaluation of the proposed hybrid mechanism demonstrates its effectiveness in high-precision motion control. Under PID control, both the long-range and hybrid stages demonstrated satisfactory decoupling performance with step and sinusoidal inputs. Transient errors attributed to the inertia of the springs and their mounting remain. Future work will assess whether the proposed two-degree-of-freedom mechanism, which houses the PEA and eliminates the return springs, reduces these errors. The Active Disturbance Rejection Control (ADRC) strategy will be investigated to deal with the existing transient errors observed during the initial and recovery phases by introducing an Extended State Observer (ESO) to treat all model uncertainties and external noise as a single synthetic noise in order to estimate and compensate directly in real time. The hybrid mechanism achieved resolutions of approximately 10 nm and 5 nm for the long-range and hybrid stages, respectively. The hybrid control strategy also reduced the rise time, settling time and overshoot relative to long-range motion actuation alone. These results validate the design’s capability for high-resolution micro/nanopositioning and confirm its potential for integration into high-performance precision systems.

## Figures and Tables

**Table 1:** Hybrid mechanism’s parameters.

Parameters	Descriptions	Value (mm)
$l_{h}$	Length of hinge	3.00
$l_{b}$	Length of beam	20.00
$d_{h}$	Distance between two hinges	5.20
*w*	Width of hinge	15.00
$t_{h}$	Thickness of hinge	0.80
$t_{b}$	Thickness of beam	8.00
*L*	Length of parallelogram beam	65.58
$t_{para}$	Thickness of parallelogram beam	0.55

**Table 2:** Descriptions of parallelogram beam parameters.

Parameters	Descriptions
*L*	Length of the parallelogram beam
*F*	Input force
*P*	Force in the X-axis
$M_{p}$	Moment
θ	Deformed angle
*h*	Height of the beam
$w_{p}$	Width of the beam

**Table 3:** Materials’ properties of Aluminum 7075.

Material Properties	Young’s Modulus	Yield Strength	Poisson’s Ratio	Density (kg/m^3^)
Value	71.7 (GPa)	503 (MPa)	0.33	2810

**Table 4:** Characteristics comparison.

Testing Methods	Amplification Ratio	Input Stiffness (N/mm)	Output Stiffness (N/mm)
Calculation	4.38	2231.81	60.18
FEA	4.23	2325.58	61.15
Difference	3.42%	4.12%	1.60%

**Table 5:** Resonant frequencies of hybrid mechanism.

Testing Methods	Long-Range Stage (Hz)	Precision Stage (Hz)
FEA	25.21	154.14
Experimental SI	25.00	148.00
Difference	0.84%	4.14%

**Table 6:** Control parameters.

Control Parameters	Long-Range Motion	Hybrid Motion
Rise time (ms)	32.40	18.90
Settling time (ms)	51.90	32.10
Overshoot (%)	24.65	5.70

**Table 7:** Performance comparison with the literature.

References	Travel Range (mm)	Resolution (nm)
[28]	1.49	≤50
[6]	0.197	40
This work	2.00	5

## Data Availability

All data supporting the findings of this study are included in the manuscript. Additional data are available from the corresponding author upon request.
